# Reduced mortality associated to cementless total hip arthroplasty in femoral neck fracture

**DOI:** 10.1038/s41598-023-43790-8

**Published:** 2023-10-06

**Authors:** Corentin Pangaud, Vanessa Pauly, Christophe Jacquet, Veronica Orleans, Laurent Boyer, Raghbir Khakha, Jean Noël Argenson, Matthieu Ollivier

**Affiliations:** 1 Institute of Movement and Locomotion, Aix-Marseille Université et CNRS 5, Marseille, France; 2 CEReSS-Health Services Research and Quality of Life Center, Faculté de Médecine, Secteur Timone, EA 3279, CEReSS -Centre D’Etude Et de Recherche Sur Les Services de Santé Et La Qualité de Vie, Aix-Marseille University, 27 Boulevard Jean Moulin, 13005 Marseille, France; 3 Department of Epidemiology and Health Economics, APHM, Marseille, France; 4 Department of Medical Information, APHM, Marseille, France; 5 Department of Trauma and Orthopaedics, Guys and St Thomas’ Hospitals, Great Maze Pond, London, SE1 9RT UK; 6 Department of Orthopedics and Traumatology, Institute of Movement and Locomotion, St. Marguerite Hospital, 270 Boulevard Sainte Marguerite, BP 29, 13274 Marseille, France

**Keywords:** Musculoskeletal system, Epidemiology, Fracture repair, Geriatrics

## Abstract

Mortality related to femoral neck fractures remains a challenging health issue, with a high mortality rate at 1 year of follow-up. Three modifiable factors appear to be under control of the surgeon: the choice of the implant, the use of cement and the timing before surgery. The aim of this research project was to study the impact on mortality each of these risk factors play during the management of femoral neck fractures. A large retrospective epidemiological study was performed using a national database of the public healthcare system. The inclusion criteria were patients who underwent joint replacement surgery after femoral neck fracture during the years 2015 to 2017. All data points were available for at least 2 years after the fracture. The primary outcome was mortality within 2 years following the surgery. We evaluated the association between mortality and the type of the implant hemiarthroplasty (HA) versus total hip arthroplasty (THA), cemented versus non cemented femoral stem as well as the timing from fracture to surgical procedure. A multivariate analysis was performed including age, gender, comorbidities/autonomy scores, social category, and obesity. We identified 96,184 patients who matched the inclusion criteria between 2015 and 2017. 64,106 (66%) patients underwent HA and 32,078 (33.4%) underwent THA. After multivariate analysis including age and comorbidities, patients who underwent surgery after 72 h intra-hospital had a higher risk of mortality: Hazard Ratio (HR) = 1.119 (1.056–1.185) *p* = 0.0001 compared to the group who underwent surgery within 24 h. THA was found to be a protective factor HR = 0.762 (0.731–0.795) *p* < 0.0001. The use of cement was correlated with higher mortality rate: HR = 1.107 (1.067–1.149) *p* < 0.0001. Three key points are highlighted by our study in the reduction of mortality related to femoral neck fracture: the use of hemiarthroplasty a surgery performed after 48 h and the use of cement for femoral stem fixation adversely affect mortality risk.

## Introduction

Femoral neck fractures are a frequent pathology seen in the elderly population^1,2^. This group of patients, often presents with multiple co-morbidities, complex social requirements and prolonged length of hospital stay, leading to an associated high mortality rate and a major healthcare cost^3^. All these factors have been pointed out in national database studies like Macaulay et al.^3^ reviewing 350,000 cases in the US population in 2006 and Bandhari et al.^4^ 280,000 for the same country in 2005. Nikitovic et al.^5^ published the cost of managing a single patient in Ontario with a femoral neck fracture as being 39,479 dollars the first year and 10,347 dollars the second year. The mortality rate after femoral neck fracture is around 30% in the 1st year following the fracture^6^.

The timing from fracture to surgery appears to be a key point in reducing mortality. In. 2010, Simunovic et al.^7^ reported a significantly lower mortality and morbidity rate in their study if surgery was performed early. The most recent study evaluating this question is Hip Attack in the Lancet in 2020^8^ which described a comparable mortality rate if the surgery was performed during the first 6 h after the trauma or twenty 4 h after.

There is strong evidence in the literature to support the use of Total Hip Arthroplasty (THA) to provide better patients reported outcomes measures (PROM’s) compared to hemiarthroplasty (HA)^9–14^. There is ongoing debate regarding the influence of the choice of the implant on the mortality rate. Wang et al.^15^ found a lower mortality rate in the THA group compared to the HA group in patients with displaced neck of femur fractures. Lewis et al.^16^ performed a meta-analysis including 1364 patients in which there was no difference in mortality rate between the two implant options. The lower mortality rate found in some studies^17^ when utilizing a THA may be explained by lower pre-operative co-morbidities levels.

Finally, the literature does not provide strong evidence on the use of cemented versus uncemented stems in femoral neck fracture surgery^18–21^. Kumar et al.^22^ did not find any difference in their meta-analysis which included 18 studies with 2819 patients, whereas Richardson et al.^23^ described a higher mortality rate for uncemented stems due to a higher reoperation rate.

The purpose of this study was to identify key points in the management of femoral neck fracture which could be “game changers” to reduce mortality^16,17^. The primary outcome was the mortality rate at 2 years of follow up after arthroplasty for femoral neck fracture. The modifiable risk factors associated to the primary outcome were analyzed: timing to surgery, the choice of implant: THA versus HA and the use of cemented or cementless femoral stems.

## Methods

A population-based cohort study was performed using the French national hospital database (*Programme de Médicalisation des Systèmes d’Information or PMSI*) covering currently 98.8% of the country population^24^*.* The PMSI contains anonymized information prospectively collected from all public and private hospitals in France for acute (PMSI-MCO) and psychiatric (PMSI-PSY) hospitalizations. Inpatient stays are converted into single diagnosis-related groups (DRGs) based on standard discharge abstracts containing administrative and clinical information: primary/secondary diagnoses, using the International Classification of Diseases, Tenth Revision (ICD-10), as well as procedural codes associated with the care provided^25,26^.

The access to the SNIIRAM is regulated and requires approval from the IDS, Institute of health data and the CNIL, the French data protection commission^27^. The study was declared for ethical considerations to the French National Data Protection Commission in accordance with the previous declaration of compliance with the reference methodology MR005- N°: 2203797.

### Outcomes

Our primary outcome was the mortality rate after arthroplasty surgery for femoral neck fracture. The mortality rate considered was the in-hospital mortality at a minimum of 2 years follow up.

Three modifiable risk factors associated to the mortality were considered:

- The time between femoral neck fracture and arthroplasty was separated in four categories: Group I for the surgery performed during the first 24 h in hospital, group II between 24 and 48 h, group III between 48 and 72 h and group IV for the patients who underwent the surgery after 72 h in hospital^8^.
- The choice of the implant: total hip arthroplasty or hemiarthroplasty^28,29^.
- The use of cemented or cementless implant for the femoral stem^23^.

The following non-modifiable risk factors of mortality were included in the multivariate analysis:

- Age
- Gender
- Comorbidities Scores^30^
- Type of hospital: Public/Private
- Obesity
- Social deprivation index^31^

### Study population

The inclusion criteria were patients hospitalized between 2015 and 2017 in France, suffering from an intracapsular femoral neck fracture according to the ICD-10 code S720 and who had undergone prosthetic surgery either with hemiarthroplasty (HA) or total hip arthroplasty (THA). Specific codes from the List of Reimbursable Products and Services (LRPS) were used to identify THA and HA and the use of cemented implants. These codes are displayed in Appendix.

The exclusion criteria were patients who suffered a contralateral femoral neck fracture within the last 2 years, patients for which it was impossible to distinguish cemented versus non-cemented implants and patients who underwent osteosynthesis.

### Population characteristics

The following demographic and clinical patients’ characteristics were extracted and computed from the database: age; gender; social deprivation assessed by the FDep09 index validated on French data^32^ categorized according to quartiles, from the least (Q1) to the most deprived areas (Q4); year of surgery; comorbidities assessed using the Elixhauser score^30^ (computed from ICD-10 codes recorded as primary or secondary diagnoses over the period of the last 12 months preceding the surgery) and obesity (from ICD 10 codes E66.x).

**Figure 1:**
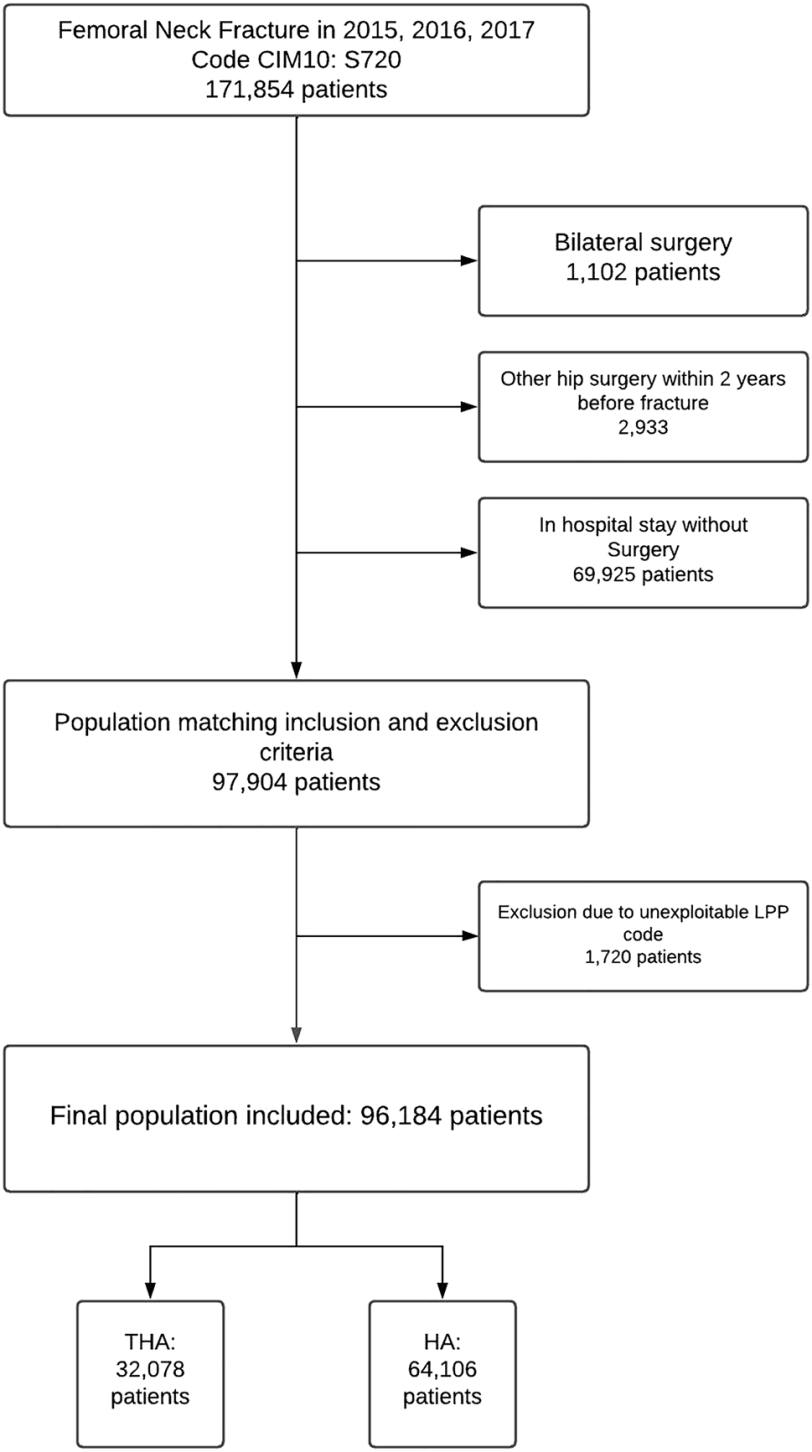
Flowchart of the study.

As a result, 96,184 patients were included during the 3 years investigated by our study. A total of 64,106 patients underwent hemiarthroplasty (66.6%) and 32,078 underwent total hip arthroplasty (33.4%) (Fig. 1). The number of women was higher than the number of men: 71,114 women (73.9%) and 25,070 men (26.1%). The HA group included 48,065 (74.9%) women and 16,041 (25.1%) men. The THA group included 23,049 (71.8%) women and 9029 (28.2%) men. The mean age was 84.7 ± 8.2 in the HA group and 76.8 ± 11.6 in the THA group. The mean number of days in hospital was 10.6 ± 6.8 in the HA group and 8.35 ± 6.02 in the THA group. The mean Charlson score was 1.27 ± 1.91. The mean Elixhauser score was 5.76 ± 7.45, and 3.71% of our population was considered as obese (BMI > 30) (Table 1).

**Table 1:** Population characteristics THA versus HA.

	HA		THA		Overall		*p* value
	N	%	N	%	N	%col	
Gender							
M	16,041	25.02	9029	28.15	25,070	26.06	< 0.0001
F	48,065	74.98	23,049	71.85	71,114	73.94	
Age							
≤ 75a	7477	11.66	13,380	41.71	20,857	21.68	< 0.0001
> 75 and < 85a	19,072	29.75	9028	28.14	28,100	29.21	
≥ 85 and < 90a	18,507	28.87	5431	16.93	23,938	24.89	
≥ 90a	19,050	29.72	4239	13.21	23,289	24.21	
Hospital category							
Public	49,682	77.5	20,454	63.76	70,136	72.92	< 0.0001
Private	14,424	22.5	11,624	36.24	26,048	27.08	
Stem fixation							
Uncemented	43,003	67.08	22,911	71.42	65,914	68.53	< 0.0001
Cemented	21,103	32.92	9167	28.58	30,270	31.47	
Year of surgery							
2015	21,326	33.27	10,093	31.46	31,419	32.67	< 0.0001
2016	21,334	33.28	10,750	33.51	32,084	33.36	
2017	21,446	33.45	11,235	35.02	32,681	33.98	
Length of stay (days)	10.7 ± 7.0		10.3 ± 6.39		10.6 ± 6.8		< 0.0001
Comorbidity score (Elixhauser)	6.26 ± 7.58		4.74 ± 7.07		5.76 ± 7.45		< 0.0001
Obesity							
Yes	2047	3.19	1380	4.3	3427	96.44	< 0.0001
No	62,059	96.81	30,698	95.7	92,757	3.56	
Overall	64,106	100	32,078	100	96,184	100	

### Statistical analysis

All the data contained a minimal period of 2 years after the femoral neck fracture at the time of data collection. Comparisons between the HA Group vs the THA Group was performed according to socio-demographics data using the Chi^2^ test for categorical data and the *t* test for comparison of means for quantitative variables.

Risk factors associated with 2-years mortality were then analyzed: We performed univariate and multivariable survival analysis using the frailty Cox model using the hospital as a random effect. Data was censored at 2 years following surgery.

Multivariate analysis was adjusted on the following variables: age (categorized), gender, Elixhauser comorbidity score (Appendix), type of hospital (academic, non-academic but public, private), obesity, year of surgery, type of implant, cemented vs non cemented implant, deprivation index, delay of surgery (categorized). No stepwise selection of variables was performed.

For each model, *p* < 0.05 was considered as statistically significant corresponding to an alpha risk α = 0.05. Analyses were performed using the SAS software ® (V9.4), SAS Institute, Cary, North Carolina, United-States.

### Ethical approval

The study was declared for ethical considerations to the French National Data Protection Commission in accordance with the previous declaration of compliance with the reference methodology MR005- N°: 2203797.

### Informed consent

The study was based only on publicly available anonymized data of the public healthcare insurance and thus no informed consent was required because these data are routinely collected, and no experimental study was made on the patients. The Institutional Review Board confirmed the absence of need for informed consent of the participant. The reference number of the IRB is MR005- N°: 2203797.

## Results

### Mortality rate

**Figure 2:**
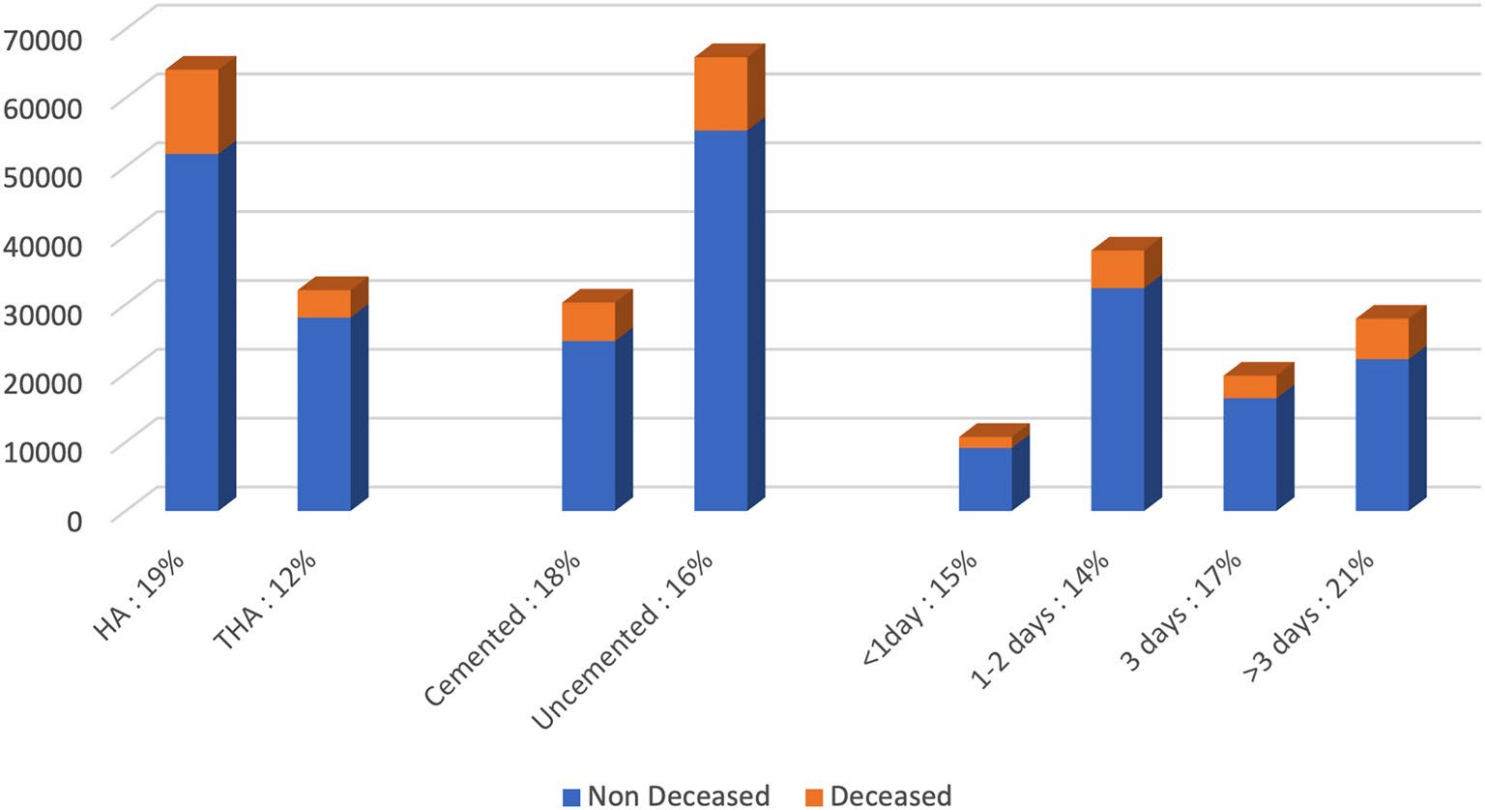
Mortality rate and number of patients in each group.

The in-hospital mortality rate was studied at 2 years of follow up: 16,238 (16.88%) patients were deceased and 79,946 (83.12%) were still alive. During the first stay, in hospital mortality associated with the femoral neck fracture was seen in 2608 (2.71%) patients (Fig. 2).

### Timing to surgery

**Figure 3:**
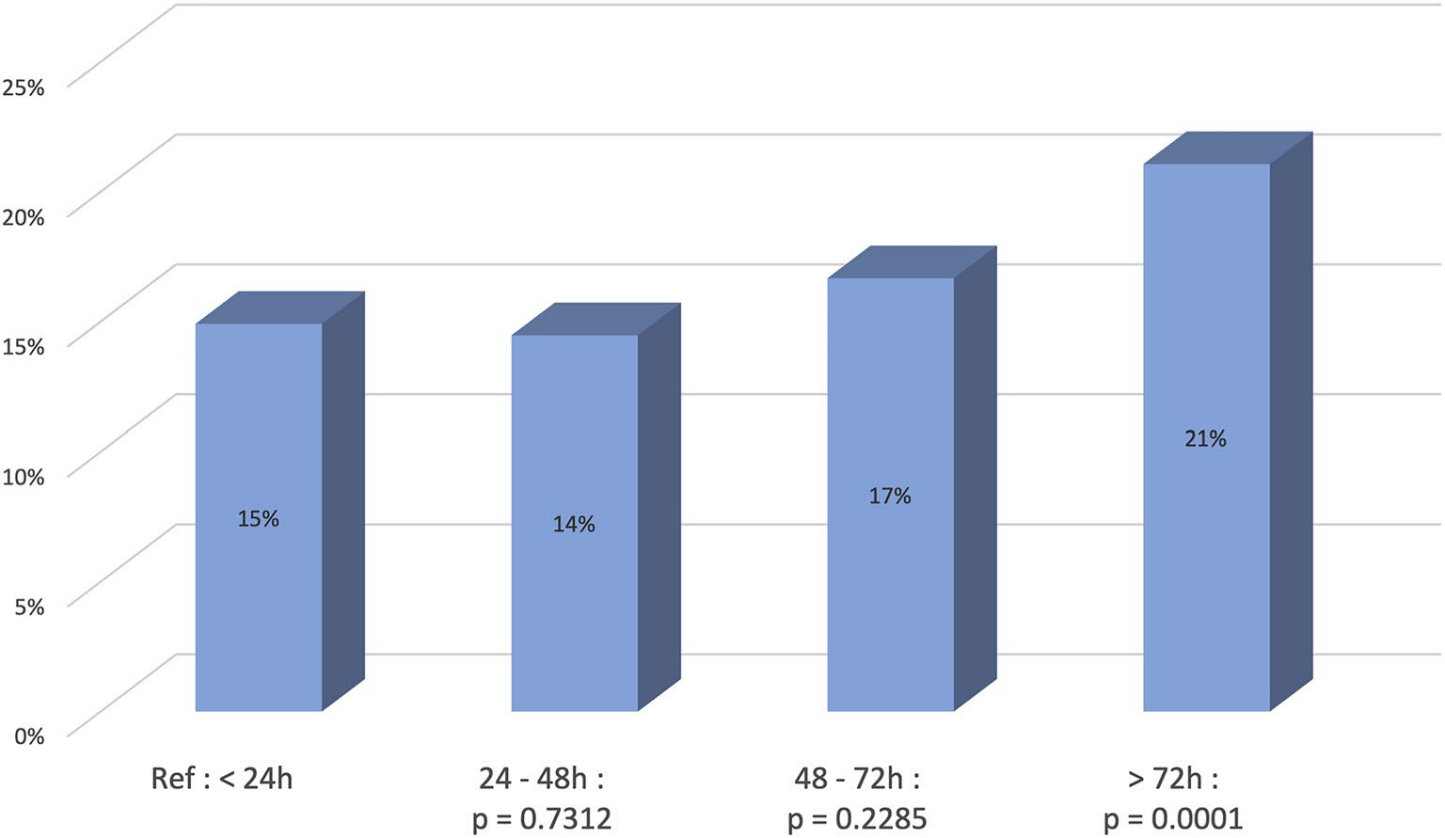
Time to surgery and mortality rate, multivariate analysis.

Group I considered those undergoing surgery for a fractured neck of femur in less than 24 h, with 10,758 patients (11.2% of total population). In this group, the 2-year mortality rate was 14.91% which was considered as the reference in the multivariate analysis. Group II looked at those undergoing surgery within 24 and 48 h, including 37,840 patients (39.3% of total population) with a mortality rate of 14.47%. Group III considered those undergoing surgery within 48 and 72 h, including 19,655 patients (20.4% of our population). In Group III the mortality rate was 16.67%. Group IV patients had surgery after 72 h, including 27,931 patients (29.1% of population) with a mortality rate of 21.06% (Fig. 3, Table 2).

**Table 2:** Population characteristics: deceased versus non-deceased.

	Non deceased patients		Deceased patients		*p* value	Overall		2-years mortality rate
	N = 79,946		N = 16,238			N = 96,184		16.88%
Age categorized	N	%	N	%	< 0.0001	N	%	
Less than 75	18,770	23.48	2087	12.85		20,857	21.68	10.01
75–85	23,862	29.85	4238	26.1		28,100	29.21	15.08
85–90	19,256	24.09	4682	28.83		23,938	24.89	19.56
> 90	18,058	22.59	5231	32.21		23,289	24.21	22.46
Age (mean ± sd)	81.53 ± 10.37		84.87 ± 8.79		< 0.0001	82.1 ± 10.2		
Gender								
Men	18,696	23.39	6374	39.25	< 0.0001	25,070	26.06	25.42
Women	61,250	76.61	9864	60.75		71,114	73.94	13.87
Social deprivation								
Favored	32,169	40.24	6428	39.59	0.0041	38,597	40.13	16.65
Disfavored	46,274	57.88	9606	59.16		55,880	58.1	17.19
Elixhauser score of comorbidity (mean ± sd)	4.89 ± 6.75		10.01 ± 9.09		< 0.0001	5.76 ± 7.45		
Obesity					< 0.0001			
No	77,123	96.47	15,634	96.28		92,757	96.44	16.85
Yes	2823	3.53	604	3.72		3427	3.56	17.62
Implant choice					< 0.0001			
HA	51,859	64.87	12,247	75.42		64,106	66.65	19.1
THA	28,087	35.13	3991	24.58		32,078	33.35	12.44
Type of implant					< 0.0001			
Non cemented	55,263	69.13	10,651	65.59		65,914	68.53	16.16
Cemented	24,683	30.87	5587	34.41		30,270	31.47	18.46
Delay of surgery from the hospital entry					< 0.0001			
Group I: < 24 h	9154	11.45	1604	9.88		10,758	11.18	14.91
Group II: 24 h-48 h	32,365	40.48	5475	33.72		37,840	39.34	14.47
Group III: 48 h-72 h	16,379	20.49	3276	20.17		19,655	20.43	16.67
Group IV: > 72 h	22,048	27.58	5883	36.23		27,931	29.04	21.06
Type of hospital					0.001			
Public	58,003	72.55	12,133	74.72		70,136	72.92	17.3
Private	21,943	27.45	4105	25.28		26,048	27.08	15.76
Year of surgery					0.2634			
2015	26,190	32.76	5229	32.2		31,419	32.67	16.64
2016	26,627	33.31	5457	33.61		32,084	33.36	17.01
2017	27,129	33.93	5552	34.19		32,681	33.98	16.99
ICU admission					< 0.0001			
No	75,183	94.04	13,897	85.58		89,080	92.61	15.6
Yes	4763	5.96	2341	14.42		7104	7.39	32.95
SAPS score for patients admitted in ICU (mean ± sd)	31.41 ± 12.24		40.35 ± 19.03		< 0.0001	34.37 ± 15.42		
Length of stay in days (mean ± sd)	10.27 ± 6.16		12.1 ± 9.19		< 0.0001	10.58 ± 6.8		

The univariate analysis found the following results: Group I: Reference, group II: HR = 0.99 (0.936–1.046) *p* = 0.7171, group III: HR = 1.109 (1.045–1.177) *p* = 0.0007, group IV: HR = 1.335 (1.263–1.411) *p* < 0.0001 (Table 3).

**Table 3:** Univariate analysis: risk factor of mortality.

	Univariate frailty Cox model			
	HR	IC95%	HR	*p* value
Age (ref: < 75)				< 0.0001
75–85	1.49	1.413	1.57	< 0.0001
85–90	2.107	2.001	2.219	< 0.0001
> 90	2.784	2.646	2.929	< 0.0001
Men versus women	1.653	1.706	1.603	< 0.0001
Disfavored versus favored	1.052	1.016	1.089	0.0041
Elixhauser score of comorbidities	1.052	1.05	1.053	< 0.0001
Obesity	0.859	0.792	0.932	0.0003
THA versus HA	0.628	0.606	0.651	< 0.0001
Cemented versus non cemented	1.108	1.073	1.145	< 0.0001
Delay of surgery in hours				< 0.0001
(Ref Group I: < 24 h)				
Group II: 24–48 h	0.99	0.936	1.046	0.7171
Group III: 48–72 h	1.109	1.045	1.177	0.0007
Group IV: > 72 h	1.335	1.263	1.411	< 0.0001
Private versus public hospital	0.925	0.893	0.958	< 0.0001
Year (ref:2015)				0.2324
2016	1.024	0.986	1.064	0.2141
2017	1.032	0.994	1.072	0.1002

The multivariate analysis including age and comorbidities only identified the Group IV to be an independent and statistically significant risk factor of mortality: HR = 1.119 (1.056–1.185) *p* = 0.0001 (Table 4).

**Table 4:** Multivariate analysis: risk Factors of mortality.

	Multivariate frailty Cox model			
	HR	IC95%	HR	*p* value
Age (ref: < 75)				< 0.0001
75–85	1.445	1.37	1.525	< 0.0001
85–90	1.996	1.891	2.106	< 0.0001
> 90	2.668	2.528	2.816	< 0.0001
Men versus women	1.6	1.549	1.652	< 0.0001
Disfavored versus favored	1.07	1.031	1.11	0.0003
Elixhauser score of comorbidities	1.048	1.046	1.05	< 0.0001
Obesity	0.958	0.882	1.041	0.3108
THA versus HA	0.765	0.734	0.798	< 0.0001
Cemented versus non cemented	1.108	1.067	1.15	< 0.0001
Delay of surgery in hours (ref: < 24 h)				< 0.0001
24–48 h	1.01	0.954	1.07	0.7312
48–72 h	1.038	0.977	1.104	0.2285
> 72 h	1.119	1.056	1.185	0.0001
Private versus public hospital	1.018	0.97	1.069	0.4589
Year (ref:2015)				0.9577
2016	1.004	0.967	1.043	0.8213
2017	1.005	0.968	1.044	0.785

### Total hip arthroplasty versus hemiarthroplasty

The HA group included 64,106 patients. At 2 years of follow-up, 12,247 (19.1%) patients were deceased and 51,859 (80.9%) were still alive. The THA group included 32,078 patients. At 2 years of follow up, 3991 (12.4%) patients were deceased and 28,087 (87.6%) were still alive (Table 2).

The univariate analysis found a Hazard Ratio (HR) = 0.628 (0.606–0651) *p* < 0.0001 for THA as a protective factor of mortality (Table 3). This was confirmed by the multivariate analysis including age and comorbidities: THA was found to be a protective factor of mortality. HR = 0.762 (0.731–0.795) *p* < 0.0001 (Table 4).

### Cement versus cementless stem

The use of cement for the femoral stem fixation was also investigated. Out of the entire cohort, 65,914 hip arthroplasties were uncemented and 30,270 were cemented. The use of cement was more frequent in the THA group: 22,911 (71.4%) versus 9167 (28.6%). In the HA group, the use of cement was also more frequent: 43,003 (67.1%) versus 21,103 (32.9). In the cemented group, 5587 (18.5%) patients were deceased at 2 years of follow up and 24,683 were still alive (81.5%) (Table 2). In the uncemented group, 10,651 (16.2%) patients were deceased at 2 years of follow up and 55,263 (83.8%) were still alive.

The univariate analysis found the following results: the use of cement was an independent risk factor of mortality at 2 years of follow up: HR = 1.108 (1.073–1.145) *p* < 0.0001 (Table 3). The multivariate analysis including age and comorbidities confirmed cement to be an independent and statistically significant risk factor of mortality: HR = 1.107 (1.067–1.149) *p* < 0.0001 (Table 4).

## Discussion

Our study highlights three crucial points in the management of femoral neck fractures: The timing from emergency admission to surgery, the choice between hemi- and total hip arthroplasty and the use of cemented or uncemented femoral stem. It appears that the right surgeon decision concerning these factors could reduce mortality. Thus, based on this large database study it appears that performing a total hip arthroplasty for femoral neck fracture with an uncemented femoral stem within 72 h after admission looks a protective attitude to reduce mortality rate.

Concerning the timing from fracture to surgery, Simunovic et al.^7^ published in their meta-analysis, an obvious reduction of mortality risk if the timing from fracture to surgery was reduced. Lewis and Waddel^33^ performed a review of the literature and found that a delay in surgery up to 48 h in ASA 1 or 2 patients did not adversely affect patient outcomes. A large randomized controlled trial was published by the Hip Attack group in the Lancet in 2020 comparing 1487 patients undergoing early surgery within 6 h after entering the hospital and 1483 patients undergoing surgery within 24 h. They did not find any significant reduction of mortality between the two groups. Our study highlights the fact that mortality really increases after 72 h as seen in Group IV: HR = 1.119 (1.056–1.185) *p* = 0.0001. This hazard ratio suggests that for every thousand surgeries for femoral neck fractures, 119 patients may die due to a prolonged delay of surgery. Another explanation for increased mortality after 72 h could be related to the taking of anti-coagulants medication creating a bias in a more vulnerable population with cardiac disease. Patients undergoing surgery in more than 72 h would be a different sub-population.

A significant reduction of mortality was found after total hip arthroplasty compared to hemiarthroplasty. This finding is consistent with two studies analyzing national data. Hansson et al.^17^ looked at a cohort of patients from the Swedish Hip Registry who found a reduction of mortality linked to THA in a population of 5815 patients. The second study was published by Wang et al.^15^ based on 70,242 American patients. They found a reduction of mortality at 2 years of follow up in a multivariate analysis: HR 1.67 (1.59–1.92). The most likely reason to explain this reduction of mortality is the increase of function after THA compared to HA^11,34–36^. Mariconda et al.^37^ described an increase of the autonomy score in the elderly after THA compared to HA. Based on these studies, it can be postulated that an arthroplasty associated with higher functional scores allows better return to autonomous living and reduced mortality. In our study the Hazard Ratio of 0.762 means that 238 patients are protected from death for every thousand surgeries if the surgeon chooses a total hip arthroplasty. Even though the population of THA and HA are difficult to compare because HA group is older and present more comorbidities than THA group. The multivariate analysis allows us to get rid of the bias concerning the differences in the populations.

There is no consensus in the literature concerning the use of cement for femoral stem fixation^19,21,23,38^. Nantha Kumar et al.^22^ performed a meta-analysis of 18 studies including 2819 patients comparing cemented versus cementless stem fixation. They found a reduced risk of intra- and post-operative peri-prosthetic fracture in the cemented arthroplasty group but no difference in terms of mortality. In comparison, Richardson et al.^23^ described an increased risk of mortality linked to the use of cementless femoral stem. In this study, they found an increased incidence of revision surgery in the cementless stem group which was associated with a higher rate of mortality. The literature review published by Chen et al.^21^ in 2018 explains that the choice of using cement or not must be based on bone quality, comorbidity profile, and age. Based on our findings, there is an increased risk of mortality with cemented stem fixation: for every thousand surgeries performed, 107 patients would potentially be saved if they have cementless fixation. Two reasons could explain the higher mortality rate associated with cement: first the physiological reaction induced by cement^39^ including hypersensitivity and lung reaction could cause death. Second, the surgeon could choose to use cement due to poor bone quality. A low bone quality could be associated with decrease overall condition. This could create a selection bias.

One potential limitation of the study stands in the only “in-hospital” mortality rate: the percentage of mortality in our series of 17% at 2 years of follow up does not includepatients who died at their home. This is why the percentage is below the results displayed in the literature of 30% of mortality at 1 year^2,6,40^. Berggren et al.^40^ published a mortality rate of 40% at 3 years of follow up after femoral neck fractures and Giummarra et al.^6^ considered 30% at 1 year, which is the most accepted percentage in the scientific community. The French National Institute of Statistics published the following results concerning places of death in 2016: 59% in-hospital mortality, 26% at home, 14% in retirement home and 1% on the roads^41^. This variation of percentage does not change the statistics and conclusions of our study even though the percentage is reduced because both groups are concerned.

The strength of the study is based on the large number of participants analyzed from a national database. The collected dataset allowed performing a powerful multivariate analysis including age and comorbidities to identify the risks factors associated to mortality following femoral neck fracture. This can bring new knowledge on the optimal timing and type of arthroplasty treatment for femoral neck fracture affecting a growing elderly population.

### Supplementary Information

Supplementary Information 1.

Supplementary Information 2.

## Data Availability

All data used for the study are available and have been uploaded on the submission platform as a filed called RESULTATSv3.docx.
