# Supplementary material for: Reduced mortality associated to cementless total hip arthroplasty in femoral neck fracture
Source: Sci Rep. 2023 Oct 6;13:16849. doi: 10.1038/s41598-023-43790-8 (PMC10558430; doi:10.1038/s41598-023-43790-8)
Supplement: Supplementary file 1 — Supplementary Information 1. [file 41598_2023_43790_MOESM1_ESM.docx]

**APPENDIX:**

***Codes Used to identify surgery:***

Total Hip Arthroplasty: NEKA010, NEKA012, NEKA013, NEKA014, NEKA015, NEKA016, NEKA017, NEKA019, NEKA020, NEKA021.

Hemiarthroplasty: NEKA011, NEKA018.

***Elixhauser comorbidity score:***

| **ELIHAUSER COMORBIDITIES** |
| --- |
|  |
|  |
| Congestive heart failure |
| Cardiac arrhythmias |
| Valvular disease |
| Pulmonary circulation disorders |
| Peripheral vascular disorders |
| Hypertension, uncomplicated |
| Hypertension, complicated |
| Paralysis |
| Other neurological disorders |
| Chronic pulmonary disease |
| Diabetes, uncomplicated |
| Diabetes, complicated |
| Hypothyroidism |
| Renal failure |
| Liver disease |
| Peptic ulcer disease, excluding bleeding |
| AIDS/HIV |
| Lymphoma |
| Metastatic cancer |
| Rheumatoid arthritis/collagen vascular diseases |
| Coagulopathy |
| Obesity |
| Weight loss |
| Fluid and electrolyte disorders |
| Blood loss anaemia |
| Deficiency anaemia |
| Acohol abuse |
| Drug abuse |
| Psychoses |
| Depression |
| Solid tumour without metastasis |
