# Supplementary material for: Reduced mortality associated to cementless total hip arthroplasty in femoral neck fracture
Source: Sci Rep. 2023 Oct 6;13:16849. doi: 10.1038/s41598-023-43790-8 (PMC10558430; doi:10.1038/s41598-023-43790-8)
Supplement: Supplementary file 2 — Supplementary Information 2. [file 41598_2023_43790_MOESM2_ESM.docx]

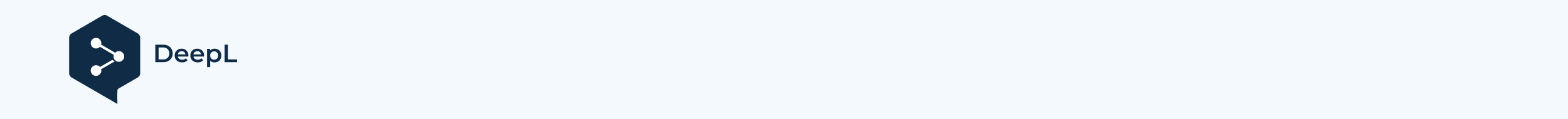


Subscribe to DeepL Pro to edit this document.
Visit [www.DeepL.com/pro](https://www.deepl.com/pro?cta=edit-document) for more information.

Research Protocol

BASE : VOBASE/PROTH _HANCHES/BASES /sejinc4.

# Objective:

Comparing the value of total hip replacement (THR) vs. intermediate hip replacement (IHR) in the management of femoral neck fractures

# Population :

All femoral neck fractures in France over 3 years: 2015, 2016, 2017 with two years of follow-up

*Step one:*

Population with cervical fracture: ICD10 code: S720

*Step 2:*

Identify patients operated on using CCAM codes for PTH and PIH

CCAM PTH codes :

NEKA010, NEKA012, NEKA013, NEKA014, NEKA015, NEKA016, NEKA017, NEKA019, NEKA020, NEKA021

CCAM PIH codes :

NEKA011, NEKA018

## Exclusion :

-> stays with a history of implant in the preceding 2 years

-> Stays for which no LOB was found to describe the type of prosthesis (cement YES / NO)

**
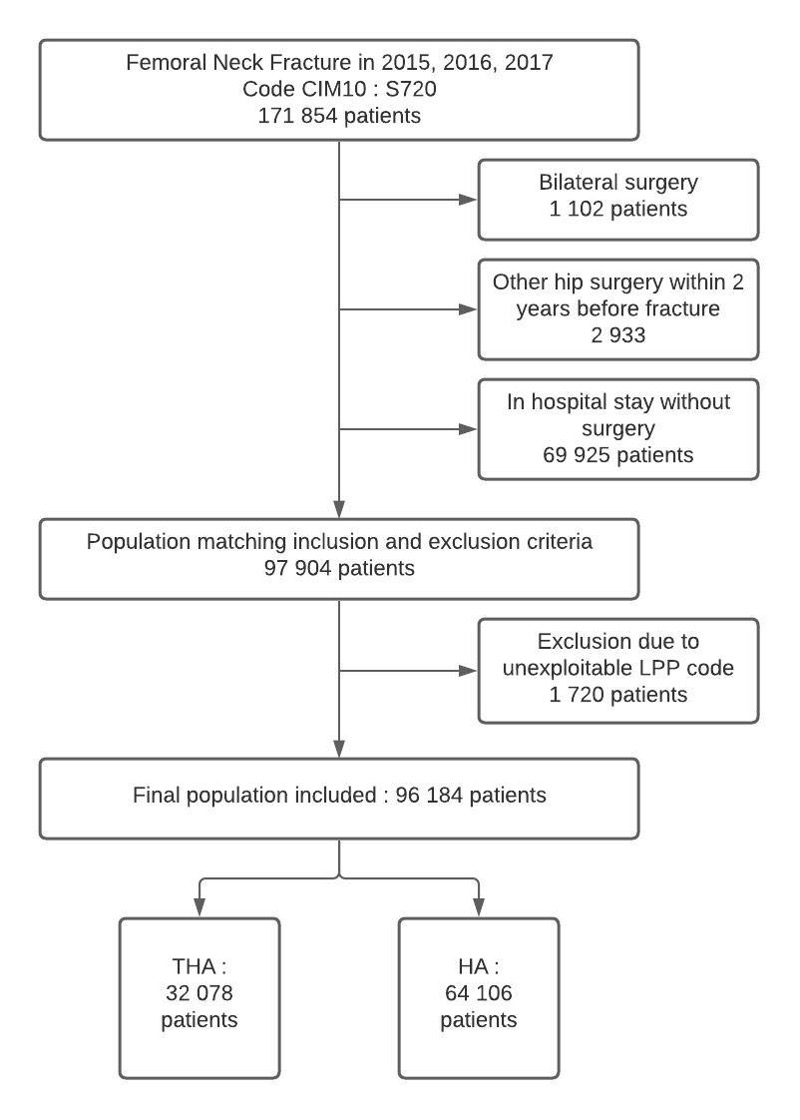
**

# Variables to be collected

**- Age: >=15 years**

**- sex**

**- cat establishment**

**- fdep: index de defav sociale**

**- dep and region of patient and facility**

**- can be the number of interventions for each plant**

**- transition to rea at sej index + igs 2**

**- comrobidities (charlson or elihauxer + weight)**

**- comorbid obesity (elihauser)**

**- identify bilaterals: how? => check with VIP if two procedures during the same stay?**

**- cement / non-cement**

## Outcomes:

Identify the following complications for the two isolated populations (PTH and PIH) and the time to complication:

- Resumptions:

NEGA001, NEGA002, NEGA003, NEGA004, NEGA005, NEKA001, NEKA002, NEKA003, NEKA004, NEKA005, NEKA006, NEKA007, NEKA008, NEKA009, NEKA022

-> Periprosthetic fracture: M966

-> Infectious complication: T84.5/ T84.6/ T84.7

-> Mechanical complications: T840/ T841/ T842/ T843/ T844

- Dislocation: ICD10 T+ codes

T840 or T849/ CCAM code for dislocation reduction NEEP002

- Transfusion: ICD Z513

- Average length of stay

- Mortality and time to onset

- Severity GHM

= > for Veronica: VAP , I would be of the opinion to go and look at the (complete) hospitalisations over 2 years and to make an analysis of the rehospitalisation stays that we have to see if we are not missing anything in the search for recoveries ....

🡪 Add survival analysis of complications

**Appendix :**

Cotyles Double Mobilités :

Analysis of LPPR codes for double mobility vs. single mobility

Lateralization :

Disappointing very few sides identified

Exclude patients operated on on both sides from the study, as it is impossible to know which side is the site of the complication

# Results

## Population included

| Initial population | cervical fracture: ICD10 code: S720 | n | 171 864 |
| --- | --- | --- | --- |
| exclusion | of which bilateral |  | 1 102 |
|  | of which other installation work before 2 years |  | 2 933 |
|  | stays without installation |  | 69 925 |
| Incidental inclusion (over 2 years) |  |  | 97 904 |
| Unreliable BVG exclusion | 1120 missing  600 LPP does not distinguish between cement and non-cement |  | 1720 |
| POP incl. final |  |  | **96 184** |

## PTH VS PIH comparison (Chi² or t test)

**Detailed file :**

### PTH VS PIH Comparison Patient characteristics + length of stay

|  | **PTH** | | | | | | **All** | |  |
| --- | --- | --- | --- | --- | --- | --- | --- | --- | --- |
|  | **0** | | | **1** | | |  |  |  |
|  | **N** | **%col** | **%l** | **N** | **%col** | **%l** | **N** | **%col** | **p value** |
| **Patient gender** | 16041 | 25.02 | 63.98 | 9029 | 28.15 | 36.02 | 25070 | 26.06 |  |
| **1 h** |  |  |  |  |  |  |  |  | <0.0001 |
| **2 f** | 48065 | 74.98 | 67.59 | 23049 | 71.85 | 32.41 | 71114 | 73.94 |  |
| **AGE** |  |  |  |  |  |  |  |  |  |
| **1_<=75a** | 7477 | 11.66 | 35.85 | 13380 | 41.71 | 64.15 | 20857 | 21.68 | <0.0001 |
| **2_>75 and <85a** | 19072 | 29.75 | 67.87 | 9028 | 28.14 | 32.13 | 28100 | 29.21 |  |
| **3_>=85 and <90a** | 18507 | 28.87 | 77.31 | 5431 | 16.93 | 22.69 | 23938 | 24.89 |  |
| **4_>=90a** | 19050 | 29.72 | 81.8 | 4239 | 13.21 | 18.2 | 23289 | 24.21 |  |
| **Detailed plant category** | 49682 | 77.5 | 70.84 | 20454 | 63.76 | 29.16 | 70136 | 72.92 | <0.0001 |
| PUBLIC |  |  |  |  |  |  |  |  |  |
| Private | 14424 | 22.5 | 55.37 | 11624 | 36.24 | 44.63 | 26048 | 27.08 |  |
| **typeCiment** | 43003 | 67.08 | 65.24 | 22911 | 71.42 | 34.76 | 65914 | 68.53 |  |
| 0 uncemented |  |  |  |  |  |  |  |  | <0.0001 |
| 1 cemented | 21103 | 32.92 | 69.72 | 9167 | 28.58 | 30.28 | 30270 | 31.47 |  |
| **Output year = base year** | 21326 | 33.27 | 67.88 | 10093 | 31.46 | 32.12 | 31419 | 32.67 |  |
| 2015 |  |  |  |  |  |  |  |  | <0.0001 |
| 2016 | 21334 | 33.28 | 66.49 | 10750 | 33.51 | 33.51 | 32084 | 33.36 |  |
| 2017 | 21446 | 33.45 | 65.62 | 11235 | 35.02 | 34.38 | 32681 | 33.98 |  |
| **All** | **64106** | **100** | **66.65** | **32078** | **100** | **33.35** | **96184** | **100** |  |
|  |  |  |  |  |  |  |  |  |  |
|  | | **PTH** | | **All** |  |  |  |  |  |
|  |  | **0** | **1** |  |  |  |  |  |  |
| **Age in years** | **N** | 64106 | 32078 | 96184 |  |  |  |  |  |
|  | **NMiss** | 0 | 0 | 0 |  |  |  |  |  |
|  | **Mean** | 84.76 | 76.78 | 82.1 | <0.0001 |  |  |  |  |
|  | **Std** | 8.2 | 11.62 | 10.2 |  |  |  |  |  |
|  | **Median** | 86 | 78 | 84 |  |  |  |  |  |
|  | **Q1** | 81 | 68 | 77 |  |  |  |  |  |
|  | **Q3** | 90 | 86 | 89 |  |  |  |  |  |
| **Fdep09: social defav index** | **N** | 63028 | 31449 | 94477 |  |  |  |  |  |
|  | **NMiss** | 1078 | 629 | 1707 | 0.91 |  |  |  |  |
|  | **Mean** | 0.07 | 0.07 | 0.07 |  |  |  |  |  |
|  | **Std** | 1.27 | 1.3 | 1.28 |  |  |  |  |  |
|  | **Median** | 0.28 | 0.25 | 0.27 |  |  |  |  |  |
|  | **Q1** | -0.5 | -0.68 | -0.54 |  |  |  |  |  |
|  | **Q3** | 0.91 | 0.93 | 0.91 |  |  |  |  |  |
|  | | | |  |  |  |  |  |  |
| **IGS 2=> only calculated when moving to REA or USC** |  |  | **PTH** | | **All** |  |  |  |  |
|  |  |  | **0** | **1** |  |  |  |  |  |
| REA_USC |  |  | 0 | 0 | 0 |  |  |  |  |
| 0 |  | **N** |  |  |  |  |  |  |  |
|  |  | **NMiss** | 59593 | 29487 | 89080 |  |  |  |  |
| 1 | **IGS2** | **N** | 4468 | 2555 | 7023 |  |  |  |  |
|  |  | **NMiss** | 45 | 36 | 81 |  |  |  |  |
|  |  | **Mean** | 36.03 | 31.47 | 34.37 |  |  |  |  |
|  |  | **Std** | 15.59 | 14.69 | 15.42 |  |  |  |  |
|  |  | **Median** | 35 | 30 | 33 |  |  |  |  |
|  |  | **Q1** | 27 | 24 | 26 |  |  |  |  |
|  |  | **Q3** | 42 | 37 | 40 |  |  |  |  |
| **Time to perform the procedure** |  |  |  |  |  |  |  |  |  |
|  |  | **PTH** |  | **All** |  |  |  |  |  |
| **Continuous** |  | **0** | **1** |  |  |  |  |  |  |
| **dlacte: time between entry into sej and realization of act in days** | **N** | 63376 | 31704 | 95080 |  |  |  |  |  |
|  | **NMiss** | 730 | 374 | 1104 |  |  |  |  |  |
|  | **Mean** | 2.27 | 2.23 | 2.26 | 0.0772 pval |  |  |  |  |
|  | **Std** | 2.79 | 2.67 | 2.75 |  |  |  |  |  |
|  | **Median** | 2 | 1 | 2 |  |  |  |  |  |
|  | **Q1** | 1 | 1 | 1 |  |  |  |  |  |
|  | **Q3** | 3 | 3 | 3 |  |  |  |  |  |
|  |  |  |  |  |  |  |  |  |  |
| **categorized** | **PTH** | | | | | | **All** |  |  |
|  | **0** | | | **1** | | |  |  |  |
|  | **N** | **%col** | **%l** | **N** | **%col** | **%l** | **N** | **%col** |  |
| **dlacte_sup3:** | 7449 | 11.62 | 69.24 | 3309 | 10.32 | 30.76 | 10758 | 11.18 |  |
| **0** |  |  |  |  |  |  |  |  |  |
| **1** | 24906 | 38.85 | 65.82 | 12934 | 40.32 | 34.18 | 37840 | 39.34 | <0.0001 |
| **2** | 12918 | 20.15 | 65.72 | 6737 | 21 | 34.28 | 19655 | 20.43 |  |
| **3 +** | 18833 | 29.38 | 67.43 | 9098 | 28.36 | 32.57 | 27931 | 29.04 |  |
| **All** | 64106 | 100 | 66.65 | 32078 | 100 | 33.35 | 96184 | 100 |  |
|  |  |  |  |  |  |  |  |  |  |
|  |  | **PTH** |  | **All** |  |  |  |  |  |
|  |  | **0** | **1** |  |  |  |  |  |  |
| **Total length of stay (in nights)** | **N** | 64106 | 32078 | 96184 |  |  |  |  |  |
|  | **NMiss** | 0 | 0 | 0 |  |  |  |  |  |
|  | **Mean** | 10.72 | 10.3 | 10.58 | p<0.0001 | to review ... |  |  |  |
|  | **Std** | 6.99 | 6.39 | 6.8 |  |  |  |  |  |
|  | **Median** | 9 | 9 | 9 |  |  |  |  |  |
|  | **Q1** | 7 | 7 | 7 |  |  |  |  |  |
|  | **Q3** | 12 | 12 | 12 |  |  |  |  |  |
|  | **PTH** | | | | | | **All** | |  |
|  | **0** | | | **1** | | |  |  |  |
|  | **N** | **%col** | **%l** | **N** | **%col** | **%l** | **N** | **%col** |  |
| REA_USC: switch to REA or USC | 59593 | 92.96 | 66.9 | 29487 | 91.92 | 33.1 | 89080 | 92.61 |  |
| 0 |  |  |  |  |  |  |  |  |  |
| 1 | 4513 | 7.04 | 63.53 | 2591 | 8.08 | 36.47 | 7104 | 7.39 | <0.0001 |

### PTH VS PIH comparison: Charlson - Elihauser comorbidities

| **comorbidity scores** |  | **PTH** | | **All** |  |
| --- | --- | --- | --- | --- | --- |
|  |  | **0** | **1** |  |  |
| **Charlson Score** | **N** | 64106 | 32078 | 96184 |  |
|  | **NMiss** | 0 | 0 | 0 |  |
|  | **Mean** | 1.36 | 1.1 | 1.27 | <0.0001 |
|  | **Std** | 1.91 | 1.89 | 1.91 |  |
|  | **Median** | 1 | 0 | 1 |  |
|  | **Q1** | 0 | 0 | 0 |  |
|  | **Q3** | 2 | 1 | 2 |  |
| **SC_elihauw_Van** | **N** | 64106 | 32078 | 96184 |  |
|  | **NMiss** | 0 | 0 | 0 |  |
|  | **Mean** | 6.26 | 4.74 | 5.76 | <0.0001 |
|  | **Std** | 7.58 | 7.07 | 7.45 |  |
|  | **Median** | 5 | 2 | 5 |  |
|  | **Q1** | 0 | 0 | 0 |  |
|  | **Q3** | 11 | 7 | 10 |  |

| **P<0.0001** | **PTH** | | | | | | **All** | |
| --- | --- | --- | --- | --- | --- | --- | --- | --- |
|  | **0** | | | **1** | | |  |  |
|  | **N** | **%col** | **%l** | **N** | **%col** | **%l** | **N** | **%col** |
| **Obesity** | 62059 | 96.81 | 66.9 | 30698 | 95.7 | 33.1 | 92757 | 96.44 |
| 0 |  |  |  |  |  |  |  |  |
| 1 | 2047 | 3.19 | 59.73 | 1380 | 4.3 | 40.27 | 3427 | 3.56 |

Details of other comorbidities fig in the **results excel** file .**..**

### PTH VS PIH comparison: outcomes

#### DC and time to death

Main outcome : **New_dc_2y_Pose: Dc within 2 years of implantation**

**19.1% in PIH vs 12.4% in PTH**

|  | **PTH** | | | | | | **All** | |  |
| --- | --- | --- | --- | --- | --- | --- | --- | --- | --- |
|  | **0** | | | **1** | | |  |  |  |
|  | **N** | **%col** | **%l** | **N** | **%col** | **%l** | **N** | **%col** |  |
| **dc_sejIndex DC for sej index** | 62101 | 96.87 | 66.36 | 31475 | 98.12 | 33.64 | 93576 | 97.29 | <0.0001 |
| 0 |  |  |  |  |  |  |  |  |  |
| 1 | 2005 | 3.13 | 76.88 | 603 | 1.88 | 23.12 | 2608 | 2.71 |  |
| **New_dc_2ans_Pose: Dc within 2 years of installation** | **51859** | **80.9** | **64.87** | **28087** | **87.56** | **35.13** | **79946** | **83.12** | **<0.0001** |
| **0** |  |  |  |  |  |  |  |  |  |
| **1** | **12247** | **19.1** | **75.42** | **3991** | **12.44** | **24.58** | **16238** | **16.88** |  |
| **DC_all: deaths over the entire period** | 48045 | 74.95 | 64.34 | 26634 | 83.03 | 35.66 | 74679 | 77.64 |  |
| 0 |  |  |  |  |  |  |  |  |  |
| 1 | 16061 | 25.05 | 74.68 | 5444 | 16.97 | 25.32 | 21505 | 22.36 |  |
| **All** | **64106** | **100** | **66.65** | **32078** | **100** | **33.35** | **96184** | **100** |  |
|  |  |  |  |  |  |  |  |  |  |
| **Delay and time of death** | | | **PTH** | | **All** |  |  |  |  |
|  |  |  | **0** | **1** |  |  |  |  |  |
| DC_all |  |  | 0 | 0 | 0 |  |  |  |  |
| 0 | **delai_Pose_dc_new** | **N** |  |  |  |  |  |  |  |
|  |  | **NMiss** | 48045 | 26634 | 74679 |  |  |  |  |
|  |  | **Mean** | . | . | . |  |  |  |  |
|  |  | **Std** | . | . | . |  |  |  |  |
|  |  | **Median** | . | . | . |  |  |  |  |
|  |  | **Q1** | . | . | . |  |  |  |  |
|  |  | **Q3** | . | . | . |  |  |  |  |
| 1 | **delai_Pose_dc_new** | **N** | 16061 | 5444 | 21505 |  |  |  |  |
|  |  | **NMiss** | 0 | 0 | 0 |  |  |  |  |
|  |  | **Mean** | 417.12 | 465.66 | 429.4 |  |  |  |  |
|  |  | **Std** | 427.08 | 436.75 | 430.06 |  |  |  |  |
|  |  | **Median** | 240 | 338 | 264 |  |  |  |  |
|  |  | **Q1** | 58 | 77 | 62 |  |  |  |  |
|  |  | **Q3** | 704 | 763 | 721 |  |  |  |  |

## A few crosses

### Cement rate trend

|  | | **Output year = base year** | | | | | | **All** | |  |
| --- | --- | --- | --- | --- | --- | --- | --- | --- | --- | --- |
|  |  | **2015** | | **2016** | | **2017** | |  |  |  |
|  |  | **N** | **PctN** | **N** | **PctN** | **N** | **PctN** | **N** | **PctN** |  |
| **PTH** | **typeCiment** | 14282 | 66.97 | 14277 | 66.92 | 14444 | 67.35 | 43003 | 67.08 |  |
| **0** | **0** |  |  |  |  |  |  |  |  | p evol =0.4017 |
|  | **1** | 7044 | 33.03 | 7057 | 33.08 | 7002 | 32.65 | 21103 | 32.92 |  |
| **1** | **0** | 7154 | 70.88 | 7666 | 71.31 | 8091 | 72.02 | 22911 | 71.42 |  |
|  | **1** | 2939 | 29.12 | 3084 | 28.69 | 3144 | 27.98 | 9167 | 28.58 | p evol =0.0656 |
| **All** | **typeCiment** | 21436 | 68.23 | 21943 | 68.39 | 22535 | 68.95 | 65914 | 68.53 |  |
|  | **0** |  |  |  |  |  |  |  |  |  |
|  | **1** | 9983 | 31.77 | 10141 | 31.61 | 10146 | 31.05 | 30270 | 31.47 | p evol =0.0464 |
|  |  |  |  |  |  |  |  |  |  |  |
|  |  |  |  |  |  |  |  |  |  |  |
| the rate of cemented prostheses tends to decrease over time | | | | |  |  |  |  |  |  |

### Evol double mob for PTH

|  | |  |  |  |  |  |  |  |  |
| --- | --- | --- | --- | --- | --- | --- | --- | --- | --- |
|  | **Output year = base year** | | | | | | **All** | |  |
|  | **2015** | | **2016** | | **2017** | |  |  |  |
|  | **N** | **%** | **N** | **%** | **N** | **%** | **N** | **%** |  |
| **typeDmob** | 8332 | 82.55 | 8931 | 83.08 | 9036 | 80.43 | 26299 | 81.98 |  |
| **0** |  |  |  |  |  |  |  |  |  |
| **1** | . | . | 32 | 0.3 | 560 | 4.98 | 592 | 1.85 | p<0.0001 |
| **9 NO categorizable** | 1761 | 17.45 | 1787 | 16.62 | 1639 | 14.59 | 5187 | 16.17 |  |
| **All** | 10093 | 100 | 10750 | 100 | 11235 | 100 | 32078 | 100 |  |
|  |  |  |  |  |  |  |  |  |  |
|  | **=> double mob appear mainly in 2017** | | | | |  |  |  |  |

### Evol Proportion public_prive

|  | | **Output year = base year** | | | | | | **All** | |  |  |
| --- | --- | --- | --- | --- | --- | --- | --- | --- | --- | --- | --- |
|  |  | **2015** | | **2016** | | **2017** | |  |  |  |  |
|  |  | **N** | **PctN** | **N** | **PctN** | **N** | **PctN** | **N** | **PctN** |  |  |
| **PTH** | **Detailed plant category** | 16371 | 76.77 | 16499 | 77.34 | 16812 | 78.39 | 49682 | 77.5 |  |  |
| **0** | **PUBLIC** |  |  |  |  |  |  |  |  |  |  |
|  | **Private** | 4955 | 23.23 | 4835 | 22.66 | 4634 | 21.61 | 14424 | 22.5 | p val trend: <0.0001 | |
| **1** | **PUBLIC** | 6422 | 63.63 | 6772 | 63 | 7260 | 64.62 | 20454 | 63.76 |  |  |
|  | **Private** | 3671 | 36.37 | 3978 | 37 | 3975 | 35.38 | 11624 | 36.24 | p val trend p=0.1174 | |
| **All** | **Detailed plant category** | 22793 | 72.55 | 23271 | 72.53 | 24072 | 73.66 | 70136 | 72.92 |  |  |
|  | **PUBLIC** |  |  |  |  |  |  |  |  |  |  |
|  | **Private** | 8626 | 27.45 | 8813 | 27.47 | 8609 | 26.34 | 26048 | 27.08 | p val trend: <0.0001 | |

=> the private sector's share is declining in 2017

=> more PTH in the private sector

### Cement*gear crossover

|  | **typeCiment** | | | | | | **All** | |
| --- | --- | --- | --- | --- | --- | --- | --- | --- |
|  | **0** | | | **1** | | |  |  |
|  | **N** | **%col** | **%l** | **N** | **%col** | **%l** | **N** | **%col** |
| **agecat** | 15282 | 23.18 | 73.27 | 5575 | 18.42 | 26.73 | 20857 | 21.68 |
| **1_<=75a** |  |  |  |  |  |  |  |  |
| **2_>75 and <85a** | 19297 | 29.28 | 68.67 | 8803 | 29.08 | 31.33 | 28100 | 29.21 |
| **3_>=85 and <90a** | 16028 | 24.32 | 66.96 | 7910 | 26.13 | 33.04 | 23938 | 24.89 |
| **4_>=90a** | 15307 | 23.22 | 65.73 | 7982 | 26.37 | 34.27 | 23289 | 24.21 |
| **All** | 65914 | 100 | 68.53 | 30270 | 100 | 31.47 | 96184 | 100 |

=> the rate of cemented prostheses increases with age

p<0.0001

### Cement * public/private

### Cement rate higher in private sector (p<0.0001; 33.5% vs 30.7% in public sector)

|  | | | **Table of typeCement by categ_detail** | | |
| --- | --- | --- | --- | --- | --- |
| **typeCiment** |  | **categ_detail(Detailed plant category)** | | | |
|  |  | **PUBLIC** | | **Private** | **Total** |
| **0** | N | 48589 | | 17325 | 65914 |
|  | % of total | 50.52 | | 18.01 | 68.53 |
|  | % l | 73.72 | | 26.28 |  |
|  | % col | 69.28 | | 66.51 |  |
| **1** | N | 21547 | | 8723 | 30270 |
|  | % of total | 22.4 | | 9.07 | 31.47 |
|  | % l | 71.18 | | 28.82 |  |
|  | % col | 30.72 | | 33.49 |  |
| **Total** |  | 70136 | | 26048 | 96184 |
|  |  | 72.92 | | 27.08 | 100 |

## Death within 2 years

### DC vs non-DC cross-tabulation (within 2 years)

=> see **TC DC 2 years ON** tab in Excel doc

|  | **PTH** | | | | | | **All** | |  |
| --- | --- | --- | --- | --- | --- | --- | --- | --- | --- |
|  | **0** | | | **1** | | |  |  |  |
|  | **N** | **%col** | **%l** | **N** | **%col** | **%l** | **N** | **%col** |  |
| **dc_sejIndex DC for sej index** | 62101 | 96.87 | 66.36 | 31475 | 98.12 | 33.64 | 93576 | 97.29 | <0.0001 |
| 0 |  |  |  |  |  |  |  |  |  |
| 1 | 2005 | 3.13 | 76.88 | 603 | 1.88 | 23.12 | 2608 | 2.71 |  |
| **New_dc_2ans_Pose: Dc within 2 years of installation** | **51859** | **80.9** | **64.87** | **28087** | **87.56** | **35.13** | **79946** | **83.12** | **<0.0001** |
| **0** |  |  |  |  |  |  |  |  |  |
| **1** | **12247** | **19.1** | **75.42** | **3991** | **12.44** | **24.58** | **16238** | **16.88** |  |

### Univariate model

Survival regression: Cox model (with Frailty model to take into account facility as a random effect and therefore intra-facility correlation).

Hazard ratio recap (here without FM as it takes too long to run in univariate mode)

| **Parameter** | **ClassVal0** | **ProbChiSq** | **HazardRatio** | **HRLowerCL** | **HRUpperCL** | **Label** |
| --- | --- | --- | --- | --- | --- | --- |
| **Age** | **(<=75 as ref)** | <.0001 |  |  |  |  |
| agecat | 2 | <.0001 | 1.49 | 1.413 | 1.57 | agecat 2 |
| agecat | 3 | <.0001 | 2.107 | 2.001 | 2.219 | agecat 3 |
| agecat | 4 | <.0001 | 2.784 | 2.646 | 2.929 | agecat 4 |
| PTH | 1 | <.0001 | 0.628 | 0.606 | 0.651 | PTH 1 |
| gender | 2 | <.0001 | 0.605 | 0.586 | 0.624 | Patient gender 2 |
| REA_USC | 1 | <.0001 | 2.001 | 1.916 | 2.091 | REA_USC: switch to REA or USC 1 |
| **Year** | 2015 as ref | 0.2324 |  |  |  |  |
| year | 2016 | 0.2141 | 1.024 | 0.986 | 1.064 | Output year = base year 2016 |
| year | 2017 | 0.1002 | 1.032 | 0.994 | 1.072 | Output year = base year 2017 |
| **dlacte_sup3** | 0 as ref | <.0001 |  |  |  |  |
| dlacte_sup3 | 1 | 0.7171 | 0.99 | 0.936 | 1.046 | dlacte_sup3: Delay between sej entry and act realization (days) recode >=3 |
| dlacte_sup3 | 2 | 0.0007 | 1.109 | 1.045 | 1.177 |  |
| dlacte_sup3 | 3 + | <.0001 | 1.335 | 1.263 | 1.411 |  |
| categ_detail | Private | <.0001 | 0.925 | 0.893 | 0.958 | Detailed establishment category Private |
| typeCiment | 1 | <.0001 | 1.108 | 1.073 | 1.145 | typeCiment 1 |
| Obesity | 1 | 0.0003 | 0.859 | 0.792 | 0.932 | Obesity 1 |
| age |  | <.0001 | 1.043 | 1.041 | 1.044 | Age in years |
| Weight_initial_1AN : charlson |  | <.0001 | 1.163 | 1.157 | 1.169 | Charlson Score |
| SC_elihauw_Van |  | <.0001 | 1.052 | 1.05 | 1.053 |  |
| Fdep09: idex defav |  | 0.0004 | 1.022 | 1.01 | 1.035 | Fdep09 - social defav index |
| duration |  | <.0001 | 1.012 | 1.011 | 1.014 | Total length of stay (in nights) |
| dlacte |  | <.0001 | 1.029 | 1.026 | 1.032 | dlacte: Delay between sej entry and act realization (days) |
| exit_deposit_time |  | <.0001 | 1.01 | 1.008 | 1.011 |  |
| IGS (only for patients transferred to REA / USC) |  | <.0001 | 1.037 | 1.034 | 1.039 | IGS2 |

### PTH vs PIH survival curve

### Multivariate model :

Survival regression: Cox model (with Frailty model to take into account facility as a random effect and therefore intra-facility correlation).

Variables introduced (no top-down strategy) :

| **PTH** |
| --- |
| **agecat** |
| **gender** |
| **SC_elihauw_Van: comorb score** |
| **categ_detail** |
| **obes** |
| **Fdep09: defav social** |
| **typeCiment** |
| **year** |
| **dlacte_sup3: dela de real de l'acte** |

| **Parameter** |  | **Pr > khi-2** | **Report** | **Confidence interval of the 95% hazard ratio** | | **Wording** |
| --- | --- | --- | --- | --- | --- | --- |
|  |  |  | **risk** |  |  |  |
| **PTH** | **1** | **<.0001** | **0.762** | **0.731** | **0.795** | **PTH 1** |
| **agecat** |  | <.0001 |  |  |  |  |
| **agecat** | **2** | <.0001 | 1.449 | 1.372 | 1.53 | agecat 2 |
| **agecat** | **3** | <.0001 | 1.997 | 1.891 | 2.109 | agecat 3 |
| **agecat** | **4** | <.0001 | 2.661 | 2.52 | 2.809 | agecat 4 |
| **gender** | **1** | <.0001 | 1.601 | 1.55 | 1.654 | Patient gender 1 |
| **SC_elihauw_Van** |  | <.0001 | 1.048 | 1.046 | 1.05 |  |
| **categ_detail** | **Private vs. public** | 0.3428 | 1.024 | 0.975 | 1.075 | Detailed establishment category Private |
| **obes** | **1** | 0.2695 | 0.954 | 0.879 | 1.037 | Obesity 1 |
| **Fdep09** |  | <.0001 | 1.039 | 1.024 | 1.054 | Fdep09 - social defav index |
| **typeCiment** | **1** | **<.0001** | **1.107** | **1.067** | **1.149** | **typeCiment 1** |
| **Year** |  | 0.934 |  |  |  |  |
| **year** | **2016** | 0.7565 | 1.006 | 0.968 | 1.046 | Output year = base year 2016 |
| **year** | **2017** | 0.7443 | 1.006 | 0.969 | 1.046 | Output year = base year 2017 |
| dlacte_sup3: Delay between sej entry and act realization (days) recode >=3 | | <.0001 |  |  |  |  |
| **dlacte_sup3** | **1** | 0.7568 | 1.009 | 0.953 | 1.069 |  |
| **dlacte_sup3** | **2** | 0.2346 | 1.038 | 0.976 | 1.104 |  |
| **dlacte_sup3** | **3** | 0.0002 | 1.116 | 1.053 | 1.183 |  |

=> less dc in PTH

=> + in cement patients

=> dc increases with age, comorbidity and disadvantage score, and time (categorized) to perform the procedure

=> no link with obesity, type of establishment and year

### **Test some interactions => to do on m.multiple**

Test interaction pth*cement => not very interesting to take into account

| Pth*cement interaction test | p=0.0003 |  |  |  |
| --- | --- | --- | --- | --- |
| **Pairwise: Chance reports for PTH** | | | |  |
| **Description** | **Point estimate** | **95% confidence interval** | |  |
| **PTH 1 vs 0 At typeCement=0** | 0.725 | 0.689 | 0.763 |  |
| **PTH 1 vs 0 At typeCement=1** | 0.835 | 0.782 | 0.892 |  |
|  |  |  |  |  |
| **Pairwise: Chance reports for typeCement** | | | |  |
| **Description** | **Point estimate** | **95% confidence interval** | |  |
| **typeCement 1 vs 0 At PTH=0** | 1.068 | 1.023 | 1.114 |  |
| **typeCement 1 vs 0 At PTH=1** | 1.23 | 1.147 | 1.318 |  |

#### Test interaction pth*agecat: p<0.0001 not very interesting to consider

| Test interaction pth*agecat |  |  |  |  | | |
| --- | --- | --- | --- | --- | --- | --- |
| **Pairwise: Chance reports for PTH** | | | |  |  |  |
| **Description** | **Point estimate: HR** | **95% confidence interval** | |  |  |  |
| **PTH 1 vs 0 At agecat=1** | 0.578 | 0.529 | 0.631 |  |  |  |
| **PTH 1 vs 0 At agecat=2** | 0.717 | 0.667 | 0.77 | => the "protective" effect of pth diminishes with age | | |
| **PTH 1 vs 0 At agecat=3** | 0.874 | 0.812 | 0.94 |  |  |  |
| **PTH 1 vs 0 At agecat=4** | 0.903 | 0.837 | 0.974 |  |  |  |
|  |  |  |  |  |  |  |
| **Pairwise: Chance reports for agecat** | | | |  |  |  |
| **Description** | **Point estimate** | **95% confidence interval** | |  |  |  |
| **agecat 2 vs 1 At PTH=0** | 1.289 | 1.203 | 1.381 |  |  |  |
| **agecat 3 vs 1 At PTH=0** | 1.701 | 1.589 | 1.821 |  |  |  |
| **agecat 4 vs 1 At PTH=0** | 2.262 | 2.115 | 2.419 |  |  |  |
| **agecat 2 vs 1 At PTH=1** | 1.599 | 1.465 | 1.746 |  |  |  |
| **agecat 3 vs 1 At PTH=1** | 2.572 | 2.35 | 2.816 |  |  |  |
| **agecat 4 vs 1 At PTH=1** | 3.533 | 3.218 | 3.879 |  |  |  |

#### Pth*categdetaol interaction test may be of interest

Because if PTH we observe more dc for private whereas NS if PIH ....

| Pth*categdetaol interaction test | p=0.0385 |  |  | can be interesting |
| --- | --- | --- | --- | --- |
| **Pairwise: Chance reports for PTH** | | | |  |
| **Description** | **Point estimate** | **95% confidence interval** | |  |
| **PTH 1 vs 0 At categ_detail=PUBLIC** | 0.74 | 0.703 | 0.779 |  |
| **PTH 1 vs 0 At categ_detail=Private** | 0.807 | 0.753 | 0.865 |  |
|  |  |  |  |  |
|  |  |  |  |  |
| **Pairwise: Chance reports for detailed establishment category** | | | |  |
| **Description** | **Point estimate** | **95% confidence interval** | |  |
| **categ_detail PUBLIC vs Private At PTH=0** | 1.004 | 0.95 | 1.062 |  |
| **categ_detail Private vs PUBLIC At PTH=0** | 0.996 | 0.942 | 1.053 |  |
| **categ_detail PUBLIC vs Private At PTH=1** | 0.92 | 0.854 | 0.993 |  |
| **categ_detail Private vs PUBLIC At PTH=1** | 1.086 | 1.007 | 1.171 | + dc for private if PTH |

| Test interaction pth*dl act | p=0.2673 |
| --- | --- |
| Test interaction pth*sex | p=0.6206 ns |
| Cement* categdetail interaction test | p=0.3747 |

### Sensitivity analysis

#### Multiple model with categorized disadvantage

Results for PTH and cement type remain unchanged (model 2 in excel doc). Below are the results for HR:

|  |  |  |  |  |  | P value | HR | 95% CI |  |  |
| --- | --- | --- | --- | --- | --- | --- | --- | --- | --- | --- |
| **Analysis of maximum likelihood estimates** | | | | | | | | | | |
| **Parameter** |  | **DDL** | **Estimated value** | **Error** | **khi-2** | **Pr > khi-2** | **Report** | **Confidence interval of the 95% hazard ratio** | | **Wording** |
|  |  |  | **parameters** | **type** |  |  | **risk** |  |  |  |
| **PTH** | **1** | 1 | -0,26784 | 0,02129 | 158,3168 | <.0001 | 0,765 | 0,734 | 0,798 | PTH 1 |
| **agecat** | **2** | 1 | 0,3683 | 0,02743 | 180,2426 | <.0001 | 1,445 | 1,37 | 1,525 | agecat 2 |
| **agecat** | **3** | 1 | 0,69099 | 0,02753 | 630,049 | <.0001 | 1,996 | 1,891 | 2,106 | agecat 3 |
| **agecat** | **4** | 1 | 0,98122 | 0,02751 | 1272,0045 | <.0001 | 2,668 | 2,528 | 2,816 | agecat 4 |
| **gender** | **1** | 1 | 0,46978 | 0,01649 | 811,7125 | <.0001 | 1,6 | 1,549 | 1,652 | Patient gender 1 |
| **SC_elihauw_Van** |  | 1 | 0,047 | 0,0008788 | 2860,6705 | <.0001 | 1,048 | 1,046 | 1,05 |  |
| **categ_detail** | **Private** | 1 | 0,01833 | 0,02475 | 0,5485 | 0,4589 | 1,018 | 0,97 | 1,069 | Detailed plant category |
| **obes** | **1** | 1 | -0,04266 | 0,04209 | 1,0275 | 0,3108 | 0,958 | 0,882 | 1,041 | Obesity 1 |
| **defav** | **1** | 1 | 0,06755 | 0,01871 | 13,035 | 0,0003 | 1,07 | 1,031 | 1,11 | defav 1 |
| **defav** | **9** | 1 | 0,10023 | 0,08221 | 1,4866 | 0,2227 | 1,105 | 0,941 | 1,299 | defav 9 |
| **typeCiment** | **1** | 1 | 0,10211 | 0,01899 | 28,9038 | <.0001 | 1,108 | 1,067 | 1,15 | typeCiment 1 |
| **year** | **2016** | 1 | 0,0044 | 0,01947 | 0,051 | 0,8213 | 1,004 | 0,967 | 1,043 | Output year = base year |
| **year** | **2017** | 1 | 0,00531 | 0,01947 | 0,0744 | 0,785 | 1,005 | 0,968 | 1,044 |  |
| **dlacte_sup3** | **1** | 1 | 0,01002 | 0,02918 | 0,118 | 0,7312 | 1,01 | 0,954 | 1,07 | dlacte_sup3: Delay between sej entry and act real (jrs) recode >=3 1 |
| **dlacte_sup3** | **2** | 1 | 0,03776 | 0,03136 | 1,4499 | 0,2285 | 1,038 | 0,977 | 1,104 |  |
| **dlacte_sup3** | **3** | 1 | 0,11206 | 0,02941 | 14,5158 | 0,0001 | 1,119 | 1,056 | 1,185 |  |

| **Effect** | **Khi-2** | **DDL** | **Pr > khi-2** | **DDL** | **Pr > khi-2** |
| --- | --- | --- | --- | --- | --- |
|  | **de Wald** |  |  | **adjusted** | **adjusted** |
| **PTH** | 158,3168 | 1 | <.0001 | 0,9006 | <.0001 |
| **agecat** | 1576,4781 | 3 | <.0001 | 2,9799 | <.0001 |
| **gender** | 811,7125 | 1 | <.0001 | 0,9955 | <.0001 |
| **SC_elihauw_Van** | 2860,6705 | 1 | <.0001 | 0,9835 | <.0001 |
| **categ_detail** | 0,5485 | 1 | 0,4589 | 0,608 | 0,2922 |
| **obes** | 1,0275 | 1 | 0,3108 | 0,9916 | 0,3081 |
| **defav** | 13,5284 | 2 | 0,0012 | 1,7027 | 0,0008 |
| **typeCiment** | 28,9038 | 1 | <.0001 | 0,8841 | <.0001 |
| **year** | 0,0843 | 2 | 0,9587 | 1,9867 | 0,9577 |
| **dlacte_sup3** | 32,7525 | 3 | <.0001 | 2,9458 | <.0001 |
| **finess** | 478,4427 | . | . | 231,33 | <.0001 |

#### Multiple model with categorized disadvantage and introduction of diabetes comorbidity

Results for PTH and cement type remain unchanged (model 3 in excel doc). Below are the results for HR:

| **Parameter** |  | **DDL** | **Estimated value** | **Error** | **khi-2** | **Pr > khi-2**  **P value** | **HR** | **95% CI** | **Wording** | |
| --- | --- | --- | --- | --- | --- | --- | --- | --- | --- | --- |
|  |  |  | **parameters** | **type** |  |  |  |  |  |  |
| **PTH** | **1** | 1 | -0,26675 | 0,02128 | 157,1292 | <.0001 | 0,766 | 0,735 | 0,798 | PTH 1 |
| **agecat** | **2** | 1 | 0,36786 | 0,02743 | 179,8397 | <.0001 | 1,445 | 1,369 | 1,524 | agecat 2 |
| **agecat** | **3** | 1 | 0,69272 | 0,02753 | 632,9883 | <.0001 | 1,999 | 1,894 | 2,11 | agecat 3 |
| **agecat** | **4** | 1 | 0,98568 | 0,02756 | 1279,2492 | <.0001 | 2,68 | 2,539 | 2,828 | agecat 4 |
| **gender** | **1** | 1 | 0,46822 | 0,0165 | 805,2924 | <.0001 | 1,597 | 1,546 | 1,65 | Patient gender 1 |
| **SC_elihauw_Van** |  | 1 | 0,04672 | 0,000885 | 2786,7668 | <.0001 | 1,048 | 1,046 | 1,05 |  |
| **categ_detail** | **Private** | 1 | 0,01858 | 0,02471 | 0,5654 | 0,4521 | 1,019 | 0,971 | 1,069 | Detailed establishment category Private |
| **obes** | **1** | 1 | -0,05571 | 0,04236 | 1,7291 | 0,1885 | 0,946 | 0,87 | 1,028 | Obesity 1 |
| **defav** | **1** | 1 | 0,06676 | 0,0187 | 12,7401 | 0,0004 | 1,069 | 1,031 | 1,109 | defav 1 |
| **defav** | **9** | 1 | 0,09624 | 0,08221 | 1,3705 | 0,2417 | 1,101 | 0,937 | 1,294 | defav 9 |
| **typeCiment** | **1** | 1 | 0,10206 | 0,01899 | 28,8966 | <.0001 | 1,107 | 1,067 | 1,149 | typeCiment 1 |
| **year** | **2016** | 1 | 0,0039 | 0,01947 | 0,0401 | 0,8413 | 1,004 | 0,966 | 1,043 | Output year = base year |
|  | **2017** | 1 | 0,00518 | 0,01946 | 0,0709 | 0,79 | 1,005 | 0,968 | 1,044 |  |
| **dlacte_sup3** | **1** | 1 | 0,01003 | 0,02918 | 0,1182 | 0,731 | 1,01 | 0,954 | 1,07 | dlacte_sup3: Delay between sej entry and act realization (days) recode >=3 |
|  | **2** | 1 | 0,03752 | 0,03136 | 1,4314 | 0,2315 | 1,038 | 0,976 | 1,104 |  |
|  | **3** | 1 | 0,11178 | 0,02941 | 14,4447 | 0,0001 | 1,118 | 1,056 | 1,185 |  |
| **comorb_diab** | **1** | 1 | 0,05828 | 0,02131 | 7,4786 | 0,0062 | 1,06 | 1,017 | 1,105 | comorb_diab 1 |

#### POP MATCH model -simple PTH vs PIH

PTH results remained unchanged in both sub-analyses.

For PTH, a sensitivity analysis was carried out on a matched population (PTH vs. non PTH) where many individuals were lost => cf doc RECAP_MATCHING_PTH_NON_PTH.docx.

**PTH VS PIH**

| **DC 2ANS** |  |  | **COX FM Univ** | | |
| --- | --- | --- | --- | --- | --- |
|  | **N** | **%** |  | P val | ***HR 95% IC*** |
| **PTH** | 1538 | 13.17 |  | **<.0001** | **0,725 [0,682 - 0,77]** |
| **PIH** | 2059 | 17.64 |  |  |  |

#### POP MATCH model -simple cement vs. non-CEMENT (n=13930 **in each group)**

For cement type, a sensitivity analysis was carried out on a matched population (cement vs. non-cement), where many individuals were lost => cf doc RECAP_MATCHING_PTH_NON_PTH.docx.

**Cement vs. non-cement**

| **DC 2ANS** |  |  | **COX FM Univ** | | |
| --- | --- | --- | --- | --- | --- |
|  | **N** | **%** |  | P val | ***HR 95% IC*** |
| **Cement** | 2505 | 17.98 |  | **0,0105** | **1.074 [1.017-1.135]** |
| **No cement** | 2349 | 16.86 |  |  |  |

## Study of recurrences within 2 years

| **Frequency** | **EventRep table by PTH** | | | |
| --- | --- | --- | --- | --- |
| **Percentage** | **eventRep** | **PTH** | | |
| **Line Pct** |  | **0** | **1** | **Total** |
| **Pct of col.** | **0** | 49663 | 26789 | 76452 |
|  |  | 51,63 | 27,85 | 79,49 |
|  |  | 64,96 | 35,04 |  |
|  |  | 77,47 | 83,51 |  |
|  | **1 recovery** | 2828 | 1519 | 4347 |
|  |  | 2,94 | 1,58 | 4,52 |
|  |  | 65,06 | 34,94 |  |
|  |  | 4,41 | 4,74 |  |
|  | **2 dc without rework** | 11615 | 3770 | 15385 |
|  |  | 12,08 | 3,92 | 16 |
|  |  | 75,5 | 24,5 |  |
|  |  | 18,12 | 11,75 |  |
|  | **Total** | 64106 | 32078 | 96184 |
|  |  | 66,65 | 33,35 | 100 |

**PTH**: 11.8% vs. 4.41% and PTH die less ....

### Cross-tabulated REPAIR VS NON REPAIR (within 2 years)

=> see **TC Rep OUINON** tab in Excel doc

### Univariate models

Competitive-risk survival regression (death as competitive evt).

There are two possible models which are complementary and do not measure exactly the same thing:

- Cox model, Cause specific (with Frailty model to take into account establishment as a random effect and therefore intra-establishment correlation).
- More commonly used for etiological purposes

which measures the instantaneous risk of recovery associated with covariates for patients who have not yet died => we provide CSHR: Cause-specific Hazard Ratio

- Fine and Gray, (with Covs sandwich estimator to take intra-establishment correlaiton into account). Based on cumulative incidences, measures the association between each covariate and the cumulative incidence (note that only the direction is interpreted, not the magnitude of the effect) = > **more commonly used for prediction purposes**

### Summary of univariate hazard ratios (**CSH** model)

| **Parameter** | **ClassVal0** | **ProbChiSq** | **HazardRatio** | **HRLowerCL** | **HRUpperCL** | **Label** |
| --- | --- | --- | --- | --- | --- | --- |
| agecat | 2 | <.0001 | 0,798 | 0,737 | 0,863 | agecat 2 |
| agecat | 3 | <.0001 | 0,766 | 0,704 | 0,833 | agecat 3 |
| agecat | 4 | <.0001 | 0,669 | 0,611 | 0,732 | agecat 4 |
| **PTH** | **1** | **0,077** | **1,063** | **0,993** | **1,138** | **PTH 1** |
| gender | 2 | 0,0188 | 0,925 | 0,866 | 0,987 | Patient gender 2 |
| REA_USC | 1 | 0,0118 | 1,147 | 1,031 | 1,277 | REA_USC: switch to REA or USC 1 |
| year | 2016 | 0,4929 | 1,026 | 0,953 | 1,104 | Output year = base year 2016 |
| year | 2017 | 0,4182 | 1,031 | 0,958 | 1,109 | Output year = base year 2017 |
| dlacte_sup3 | 1 | 0,0798 | 1,097 | 0,989 | 1,218 | dlacte_sup3: Delay between sej entry and act realization (days) recode >=3 |
| dlacte_sup3 | 2 | 0,475 | 0,959 | 0,854 | 1,076 |  |
| dlacte_sup3 | 3 | 0,3332 | 0,947 | 0,849 | 1,057 |  |
| categ_detail | Private | <.0001 | 1,258 | 1,159 | 1,366 | Detailed establishment category Private |
| **typeCiment** | **1** | **0,4235** | **0,971** | **0,904** | **1,043** | **typeCiment 1** |
| obes | 1 | 0,0864 | 1,134 | 0,982 | 1,309 | Obesity 1 |
| age |  | <.0001 | 0,984 | 0,981 | 0,987 | Age in years |
| Initial_Weight_1AN | | <.0001 | 0,968 | 0,953 | 0,983 | Charlson Score |
| SC_elihauw_Van | | 0,0007 | 0,993 | 0,989 | 0,997 |  |
| Fdep09 |  | 0,1926 | 0,982 | 0,955 | 1,009 | Fdep09 - social defav index |
| duration |  | <.0001 | 1,015 | 1,012 | 1,017 | Total length of stay (in nights) |
| dlacte |  | 0,0842 | 0,99 | 0,979 | 1,001 | dlacte: Delay between sej entry and act realization (days) |
| exit_deposit_time | | <.0001 | 1,017 | 1,015 | 1,02 |  |
| IGS |  | 0,0083 | 0,99 | 0,982 | 0,997 | IGS2 |

For details of the elihauser and Charlson comorb, see the **HR UNIV 2** tab in the excel doc on trade-ins.

### Multivariate model :

We present the results of two survival models with competitive risks

- CSH and SDH

Adjustment variables :

| **PTH** |
| --- |
| **agecat** |
| **gender** |
| **SC_elihauw_Van: comorb score** |
| **categ_detail** |
| **obes** |
| **Fdep09: defav social** |
| **typeCiment** |
| **year** |
| **dlacte_sup3 : dela de real of the act** |

In yellow: divergent results between the two methods CSH vs SDH (Fine an Gray)

|  | **CSH (instant risk)** | | | | | |  | ***Fine and Gray (cumulative incidence risk)*** | | | |
| --- | --- | --- | --- | --- | --- | --- | --- | --- | --- | --- | --- |
| **Wording** | **Parameter** |  | **Pr > khi-2** | **Report** | **Confidence interval of the 95% hazard ratio** | |  | ***Pr > khi-2*** | ***Report*** | ***Confidence interval of the 95% hazard ratio*** | |
|  |  |  |  | **risk** |  |  |  |  | ***risk*** |  |  |
| **PTH 1** | **PTH** | **1** | 0,0168 | 0,914 | 0,849 | 0,984 |  | ***0,3109*** | ***0,959*** | ***0,884*** | ***1,04*** |
| **agecat 2** | **agecat** | **2** | <.0001 | 0,793 | 0,73 | 0,861 |  | *<.0001* | *0,781* | *0,718* | *0,848* |
| **agecat 3** | **agecat** | **3** | <.0001 | 0,765 | 0,699 | 0,836 |  | *<.0001* | *0,735* | *0,671* | *0,804* |
| **agecat 4** | **agecat** | **4** | <.0001 | 0,668 | 0,606 | 0,736 |  | *<.0001* | *0,617* | *0,557* | *0,683* |
| **Patient gender 1** | **gender** | **1** | 0,0515 | 1,069 | 1 | 1,143 |  | *0,4948* | *1,024* | *0,957* | *1,096* |
|  | **SC_elihauw_Van** |  | 0,0228 | 0,995 | 0,991 | 0,999 |  | *<.0001* | *0,99* | *0,986* | *0,994* |
| **Detailed establishment category Private** | **categ_detail** | **Private** | <.0001 | 1,267 | 1,163 | 1,38 |  | *<.0001* | *1,273* | *1,165* | *1,391* |
| **Obesity 1** | **obes** | **1** | 0,2856 | 1,082 | 0,936 | 1,252 |  | *0,2901* | *1,079* | *0,937* | *1,242* |
| **Fdep09 - social defav index** | **Fdep09** |  | 0,4262 | 0,989 | 0,962 | 1,016 |  | *0,8387* | *0,997* | *0,966* | *1,029* |
| **typeCiment 1** | **typeCiment** | **1** | 0,5256 | 0,977 | 0,909 | 1,05 |  | *0,2432* | *0,947* | *0,865* | *1,038* |
| **Year of release** | **year** | **2016** | 0,5607 | 1,022 | 0,949 | 1,101 |  | *0,5257* | *1,026* | *0,949* | *1,109* |
|  | **year** | **2017** | 0,3929 | 1,033 | 0,959 | 1,112 |  | *0,3925* | *1,033* | *0,959* | *1,114* |
| **dlacte_sup3: Delay between sej entry and act real (jrs) recode** | **dlacte_sup3** | **1** | 0,1115 | 1,089 | 0,981 | 1,209 |  | *0,107* | *1,088* | *0,982* | *1,206* |
|  | **dlacte_sup3** | **2** | 0,4706 | 0,958 | 0,853 | 1,076 |  | *0,4579* | *0,957* | *0,854* | *1,074* |
|  | **dlacte_sup3** | **3** | 0,4187 | 0,955 | 0,855 | 1,067 |  | *0,2761* | *0,941* | *0,842* | *1,05* |

Results for PTH differ according to which method is used:

PIH is associated with a higher instantaneous risk of revision (for patients who have not yet died) than PTH, but this does not increase the incidence of revision within 2 years of placement (because patients actually die more).

For the other factors, the results converge between the two methods ....

### Test of some interactions (on multiple model, CSH )

#### categ_detail*PTH, p=0.56

#### PTH*cement type, **p=0.028**

| **PTH*cement type** |  |  |  |  |  |
| --- | --- | --- | --- | --- | --- |
| **Type 3 tests** | | | | | |
| **Effect** | **Khi-2** | **DDL** | **Pr > khi-2** | **DDL** | **Pr > khi-2** |
|  | **de Wald** |  |  | **adjusted** | **adjusted** |
| **PTH** | 9,9889 | 1 | 0,0016 | 0,9147 | 0,0013 |
| **agecat** | 72,1332 | 3 | <.0001 | 2,981 | <.0001 |
| **gender** | 3,8472 | 1 | 0,0498 | 0,9971 | 0,0496 |
| **SC_elihauw_Van** | 5,3478 | 1 | 0,0207 | 0,9883 | 0,0204 |
| **categ_detail** | 28,9411 | 1 | <.0001 | 0,6429 | <.0001 |
| **obes** | 1,0852 | 1 | 0,2975 | 0,9922 | 0,2951 |
| **Fdep09** | 0,6622 | 1 | 0,4158 | 0,8561 | 0,3604 |
| **typeCiment** | 3,0632 | 1 | 0,0801 | 0,903 | 0,0696 |
| **year** | 0,7618 | 2 | 0,6833 | 1,99 | 0,6809 |
| **dlacte_sup3** | 15,372 | 3 | 0,0015 | 2,9514 | 0,0014 |
| **PTH*cement type** | 4,7268 | 1 | 0,0297 | 0,9629 | 0,028 |
| **finess** | 352,8426 | . | . | 197,19 | <.0001 |
|  |  |  |  |  |  |
| **Pairwise: Chance reports for PTH** | | | |  |  |
| **Description** | **Point estimate** | **95% confidence interval** | |  |  |
| **PTH 1 vs 0 At typeCement=0** | 0,87 | 0,798 | 0,948 |  | **=> the interaction shows that, in reality, the protective effect of PTH on revision only occurs when the prosthesis is not cemented.** |
| **PTH 1 vs 0 At typeCement=1** | 1,017 | 0,901 | 1,148 | NS |  |
|  |  |  |  |  |  |
| **Pairwise: Chance reports for typeCement** | | | |  |  |
| **Description** | **Point estimate** | **95% confidence interval** | |  |  |
| **typeCement 1 vs 0 At PTH=0** | 0,924 | 0,846 | 1,01 | NS | p=0.08 |
| **typeCement 1 vs 0 At PTH=1** | 1,081 | 0,963 | 1,213 | NS | p=0,17 |

**>> less revision for PTH vs PIH if prosthesis not cemented.**

**>> if the prosthesis is cemented, no significant difference**

#### PTH*agecat , p=0.0491

| **PTH*agecat** |  |  |  |  |  |  |  |
| --- | --- | --- | --- | --- | --- | --- | --- |
| **Type 3 tests** | | | | | |  |  |
| **Effect** | **Khi-2** | **DDL** | **Pr > khi-2** | **DDL** | **Pr > khi-2** |  |  |
|  | **de Wald** |  |  | **adjusted** | **adjusted** |  |  |
| **PTH** | 5,662 | 1 | 0,0173 | 0,9754 | 0,0166 |  |  |
| **agecat** | 19,6476 | 3 | 0,0002 | 2,9716 | 0,0002 |  |  |
| **gender** | 3,901 | 1 | 0,0483 | 0,9971 | 0,048 |  |  |
| **SC_elihauw_Van** | 5,6539 | 1 | 0,0174 | 0,9887 | 0,0171 |  |  |
| **categ_detail** | 28,4761 | 1 | <.0001 | 0,6462 | <.0001 |  |  |
| **obes** | 1,1477 | 1 | 0,284 | 0,9923 | 0,2817 |  |  |
| **Fdep09** | 0,6691 | 1 | 0,4134 | 0,8568 | 0,3584 |  |  |
| **typeCiment** | 0,424 | 1 | 0,515 | 0,8971 | 0,4717 |  |  |
| **year** | 0,7799 | 2 | 0,6771 | 1,9901 | 0,6747 |  |  |
| **dlacte_sup3** | 15,2495 | 3 | 0,0016 | 2,9516 | 0,0015 |  |  |
| **PTH*agecat** | 7,8183 | 3 | 0,0499 | 2,9787 | 0,0491 |  |  |
| **finess** | 346,0123 | . | . | 195,16 | <.0001 |  |  |
|  |  |  |  |  |  |  |  |
| **Pairwise: Chance reports for PTH** | | | |  |  |  |  |
| **Description** | **Point estimate** | **95% confidence interval** | |  |  |  |  |
| **PTH 1 vs 0 At agecat=1** | 0,862 | 0,763 | 0,974 |  | weights the protective effect of PTH, only significant for classes 1 and 2 (<=85 years) | | |
| **PTH 1 vs 0 At agecat=2** | 0,851 | 0,753 | 0,961 |  |  |  |  |
| **PTH 1 vs 0 At agecat=3** | 0,995 | 0,858 | 1,152 | ns |  |  |  |
| **PTH 1 vs 0 At agecat=4** | 1,094 | 0,918 | 1,302 | ns |  |  |  |
|  |  |  |  |  |  |  |  |
| **Pairwise: Chance reports for agecat** | | | |  |  |  |  |
| **Description** | **Point estimate** | **95% confidence interval** | | bof => the effects of age are more marked for HSPs | | | |
| **agecat 2 vs 1 At PTH=0** | 0,783 | 0,699 | 0,876 |  |  |  |  |
| **agecat 3 vs 1 At PTH=0** | 0,724 | 0,645 | 0,814 |  |  |  |  |
| **agecat 4 vs 1 At PTH=0** | 0,623 | 0,552 | 0,703 |  |  |  |  |
| **agecat 2 vs 1 At PTH=1** | 0,772 | 0,682 | 0,875 |  |  |  |  |
| **agecat 3 vs 1 At PTH=1** | 0,835 | 0,721 | 0,968 |  |  |  |  |
| **agecat 4 vs 1 At PTH=1** | 0,79 | 0,665 | 0,938 |  |  |  |  |
|  |  |  |  |  |  |  |  |
| PTH*dlacte_sup3 | NS, p=0.0623 |  |  |  |  |  |  |
| PTH*sex | NS, p=0.0782 |  |  |  |  |  |  |
| categ_det*typeCement | NS , p=0,054 |  |  |  |  |  |  |

### The different types of trade-in

Selection of patients with recovery within 2 years

**PTH vs PIH comparison**

Mechanical complications account for the majority of rework etiologies.

Fewer revisions for infectious complications in PTH vs PIH and more for fractures

|  | **PIH** | | **PTH** | | | | **Total** |  | |  |
| --- | --- | --- | --- | --- | --- | --- | --- | --- | --- | --- |
|  | **N** | %col |  | **N** | %col |  | **N** | **%col** | |  |
| **FrperiProth** | **348** | **12,31** |  | **227** | **14,94** |  | **575** | **13,23** | |  |
| **ComplInfect** | **724** | **25,6** |  | **307** | **20,21** |  | **1031** | **23,72** | |  |
| **ComplMec** | **1709** | **60,43** |  | **921** | **60,63** |  | **2630** | **60,5** | |  |
|  |  |  |  |  |  |  |  |  | |  |
| **Cement vs. non-cement comparisons** | | | | | | | | |  |  |
|  | No cement | | **cement** | | Total trade-in |  |  |  |  |  |
|  | **N** | %col | **N** | %col | **N** | **%col** |  |  |  |  |
| **FrperiProth** | **441** | **14,68** | **134** | **9,98** | **575** | **13,23** |  |  |  |  |
| **ComplInfect** | **694** | **23,1** | **337** | **25,09** | **1031** | **23,72** |  |  |  |  |
| **ComplMec** | **1795** | **59,75** | **835** | **62,17** | **2630** | **60,5** |  |  |  |  |
|  |  |  |  |  |  |  |  |  | |  |

=> less periproth fractures for cement

=> no more complications with infection, mechanics and cement dislocation

Nb: % represents the proportion of repeat cases for a given cause among all patients with repeat cases.

Revision rates by etiology

|  | **PIH** | | **PTH** | | **global** |  |  |  | **Uncemented** | | **Cemented** | | **All** |  |
| --- | --- | --- | --- | --- | --- | --- | --- | --- | --- | --- | --- | --- | --- | --- |
|  | **N** | **%** | **N** | **%** | **N** | **%** |  |  | **N** | **%** | **N** | **%** | **N** | **%** |
| **FrperiProth** | **348** | 0,5 | **227** | 0,7 | **575** | **0,6** |  | **FrperiProth** | **441** | 0,7 | **134** | 0,4 | **575** | **0,6** |
| **ComplInfect** | **724** | 1,1 | **307** | 1 | **1031** | **1,1** |  | **ComplInfect** | **694** | 1,1 | **337** | 1,1 | **1031** | **1,1** |
| **ComplMec** | **1709** | 2,7 | **921** | 2,9 | **2630** | **2,7** |  | **ComplMec** | **1795** | 2,7 | **835** | 2,8 | **2630** | **2,7** |

#### Results of univariate and multivariate statistical models by type of recovery

Survival models with CR (CSH model)

1. **Revision for mechanical complications (within 2 years)**

**Univariate stat (only pth and cement as factors)**

|  | **Pr > khi-2** | **Report** | **Confidence interval of the 95% hazard ratio** | | **Wording** |
| --- | --- | --- | --- | --- | --- |
|  |  | **risk** |  |  |  |
|  | 0,2487 | 1,053 | 0,965 | 1,149 | PTH 1 |
|  | **Pr > khi-2** | **Report** | **Confidence interval of the 95% hazard ratio** | | **Wording** |
|  |  | **risk** |  |  |  |
|  | 0,515 | 1,031 | 0,941 | 1,13 | typeCiment 1 |
| **Multivariate stat** | | | | | |
|  | **P value** | **Report** | **Confidence interval of the 95% hazard ratio** | | **Wording** |
|  |  |  |  |  |  |
|  |  | **risk** |  |  |  |
|  | 0,0107 | 0,884 | 0,804 | 0,972 | PTH 1 |
|  | <.0001 | 0,784 | 0,705 | 0,871 | agecat 2 |
|  | <.0001 | 0,696 | 0,62 | 0,782 | agecat 3 |
|  | <.0001 | 0,645 | 0,57 | 0,73 | agecat 4 |
|  | 0,736 | 0,985 | 0,902 | 1,075 | Patient gender 1 |
|  | 0,0014 | 0,991 | 0,986 | 0,997 |  |
|  | <.0001 | 1,385 | 1,243 | 1,543 | Detailed establishment category Private |
|  | 0,6514 | 0,956 | 0,785 | 1,163 | Obesity 1 |
|  | 0,6888 | 0,993 | 0,958 | 1,028 | Fdep09 - social defav index |
|  | 0,4014 | 1,04 | 0,949 | 1,14 | typeCiment 1 |
|  | 0,3737 | 1,044 | 0,95 | 1,148 | Output year = base year 2016 |
|  | 0,8727 | 1,008 | 0,916 | 1,109 | Output year = base year 2017 |
|  | 0,0165 | 1,182 | 1,031 | 1,354 | dlacte_sup3: Delay between sej entry and act realization (days) recode >=3 1 |
|  | 0,6206 | 1,039 | 0,893 | 1,208 | dlacte_sup3: Delay between sej entry and act realization (days) recode >=3 2 |
|  | 0,5933 | 0,961 | 0,831 | 1,112 | dlacte_sup3: Delay between sej entry and act realization (days) recode >=3 3 |

In multivariate analysis, more rework for PIH vs PTH; no difference according to cement.

1. **Fracture revision (within 2 years)**

| **Univariate stat (only pth and cement as factors)** | | | | | | | | | | |
| --- | --- | --- | --- | --- | --- | --- | --- | --- | --- | --- |
|  | **Pr > khi-2** | | **Report** | | **Confidence interval of the 95% hazard ratio** | | | | **Wording** | |
|  |  |  | **risk** | |  |  |  |  |  |  |
|  | 0,0079 | | 1,278 | | 1,066 | | 1,531 | | PTH 1 | |
|  | **Pr > khi-2** | | **Report** | | **Confidence interval of the 95% hazard ratio** | | | | **Wording** | |
|  |  |  | **risk** | |  |  |  |  |  |  |
|  | 0,0002 | | 0,665 | | 0,539 | | 0,822 | | typeCiment 1 | |
| **Multivariate stat** | | | | | | | | | | |
| **Pr > khi-2** | | **Report** | | **Confidence interval of the 95% hazard ratio** | | | | **Wording** | |  |
|  |  | **risk** | |  |  |  |  |  |  |  |
| 0,0215 | | 1,263 | | 1,035 | | 1,542 | | PTH 1 | |  |
| 0,6687 | | 1,053 | | 0,831 | | 1,335 | | agecat 2 | |  |
| 0,4097 | | 1,112 | | 0,863 | | 1,433 | | agecat 3 | |  |
| 0,6368 | | 1,067 | | 0,816 | | 1,395 | | agecat 4 | |  |
| 0,3123 | | 0,906 | | 0,748 | | 1,097 | | Patient gender 1 | |  |
| 0,0098 | | 0,984 | | 0,972 | | 0,996 | | SC_elihauw_Van | |  |
| 0,6074 | | 1,063 | | 0,841 | | 1,344 | | Detailed establishment category Private | |  |
| 0,4136 | | 1,174 | | 0,799 | | 1,726 | | Obesity 1 | |  |
| 0,6744 | | 0,984 | | 0,911 | | 1,062 | | Fdep09 - social defav index | |  |
| 0,0002 | | 0,668 | | 0,541 | | 0,826 | | typeCiment 1 | |  |
| 0,2923 | | 1,12 | | 0,907 | | 1,382 | | Output year = base year 2016 | |  |
| 0,0101 | | 1,305 | | 1,065 | | 1,6 | | Output year = base year 2017 | |  |
| 0,4041 | | 0,892 | | 0,682 | | 1,167 | | dlacte_sup3: Delay between sej entry and act realization (days) recode >=3 1 | |  |
| 0,0625 | | 0,747 | | 0,55 | | 1,015 | | dlacte_sup3: 3 2 | |  |
| 0,2182 | | 0,836 | | 0,628 | | 1,112 | | dlacte_sup3: 3 | |  |

In multivariate analysis, there were more repeat fractures in THPs and fewer in cemented THPs.

1. **Resumption for infection (within 2 years)**

| **Univariate stat (only pth and cement as factors)**   \|  \| **Pr > khi-2** \| **Report** \| **Confidence interval of the 95% hazard ratio** \| \| **Wording** \| \| --- \| --- \| --- \| --- \| --- \| --- \| \|  \| **risk** \| \|  \| 0,0447 \| 0,864 \| 0,749 \| 0,997 \| PTH 1 \| \|  \| **Pr > khi-2** \| **Report** \| **Confidence interval of the 95% hazard ratio** \| \| **Wording** \| \|  \| **risk** \| \|  \| 0,8141 \| 1,017 \| 0,881 \| 1,174 \| typeCiment 1 \| \| **Multivariate stat** \| **Pr > khi-2** \| **Report** \| **Confidence interval of the 95% hazard ratio** \| \| **Wording** \| \|  \| **risk** \| \|  \| <.0001 \| 0,728 \| 0,623 \| 0,851 \| PTH 1 \| \|  \| <.0001 \| 0,665 \| 0,562 \| 0,789 \| agecat 2 \| \|  \| 0,0002 \| 0,709 \| 0,593 \| 0,848 \| agecat 3 \| \|  \| <.0001 \| 0,575 \| 0,473 \| 0,701 \| agecat 4 \| \|  \| <.0001 \| 1,419 \| 1,246 \| 1,616 \| Patient gender 1 \| \|  \| 0,1124 \| 1,006 \| 0,999 \| 1,014 \|  \| \|  \| 0,7024 \| 0,966 \| 0,808 \| 1,154 \| Detailed establishment category Private \| \|  \| 0,0011 \| 1,526 \| 1,185 \| 1,967 \| Obesity 1 \| \|  \| 0,9201 \| 0,997 \| 0,943 \| 1,055 \| Fdep09 - social defav index \| \|  \| 0,6713 \| 1,032 \| 0,892 \| 1,193 \| typeCiment 1 \| \|  \| 0,8108 \| 0,982 \| 0,843 \| 1,143 \| Release year = 2016 \| \|  \| 0,9182 \| 1,008 \| 0,867 \| 1,172 \| Release year = 2017 \| \|  \| 0,7278 \| 1,041 \| 0,83 \| 1,305 \| dlacte_sup3: Delay between sej entry and act realization (days) recode >=3 1 \| \|  \| 0,7314 \| 1,044 \| 0,817 \| 1,335 \| dlacte_sup3: 2 \| \|  \| 0,044 \| 1,265 \| 1,006 \| 1,591 \| dlacte_sup3: 3 \| |
| --- | --- | --- | --- | --- | --- | --- | --- | --- | --- | --- | --- | --- | --- | --- | --- | --- | --- | --- | --- | --- | --- | --- | --- | --- | --- | --- | --- | --- | --- | --- | --- | --- | --- | --- | --- | --- | --- | --- | --- | --- | --- | --- | --- | --- | --- | --- | --- | --- | --- | --- | --- | --- | --- | --- | --- | --- | --- | --- | --- | --- | --- | --- | --- | --- | --- | --- | --- | --- | --- | --- | --- | --- | --- | --- | --- | --- | --- | --- | --- | --- | --- | --- | --- | --- | --- | --- | --- | --- | --- | --- | --- | --- | --- | --- | --- | --- | --- | --- | --- | --- | --- | --- | --- | --- | --- | --- | --- | --- | --- | --- | --- | --- | --- | --- | --- | --- | --- | --- | --- | --- | --- | --- | --- | --- | --- | --- |

In multivariate analysis, we observed more fracture revision in PIH and no difference for cements.

## Study of dislocations within 2 years

Crosstabulation, event type * prosthesis type

| **Frequency** | **eventLUX table by PTH** | | | |
| --- | --- | --- | --- | --- |
| **Percentage** | **eventLUX** | **PTH** | | |
| **Line Pct** |  | **0** | **1** | **Total** |
| **Pct of col.** | **0** | 49075 | 26467 | 75542 |
|  |  | 51,02 | 27,52 | 78,54 |
|  |  | 64,96 | 35,04 |  |
|  |  | 76,55 | 82,51 |  |
|  | **1 dislocation** | 3647 | 1904 | 5551 |
|  |  | 3,79 | 1,98 | 5,77 |
|  |  | 65,7 | 34,3 |  |
|  |  | 5,69 | 5,94 |  |
|  | **2 dc without dislocation** | 11384 | 3707 | 15091 |
|  |  | 11,84 | 3,85 | 15,69 |
|  |  | 75,44 | 24,56 |  |
|  |  | 17,76 | 11,56 |  |
|  | **Total** | 64106 | 32078 | 96184 |
|  |  | 66,65 | 33,35 | 100 |

**>>** there are slightly more PTH dislocations, but at the same time fewer deaths, so a lower probability of dislocation.

### LUXATION VS NON LUXATION cross-tabulation (within 2 years)

=> see **TC Complic Lux YES_NO** tab in Excel doc on dislocations.

### Univariate models

Survival regression with competitive risks (death as a competitive event) => same method and remarks as for the analysis of recoveries.

There are two possible models which are complementary and do not measure exactly the same thing:

- Cox model, Cause specific (with Frailty model to take into account establishment as a random effect and therefore intra-establishment correlation).
- More commonly used for etiological purposes

which measures the instantaneous risk of recovery associated with covariates for patients who have not yet died => we provide CSHR: Cause-specific Hazard Ratio

- Fine and Gray, (with Covs sandwich estimator to take intra-establishment correlation into account). Based on cumulative incidences, measures the association between each covariate and the cumulative incidence (note that only the direction is interpreted, not the magnitude of the effect) = > **more commonly used for prediction.**

#### Summary of univariate hazard ratios (**CSH** model)

| **Parameter** | **ClassVal0** | **ProbChiSq** | **HazardRatio** | **HRLowerCL** | **HRUpperCL** | **Label** |
| --- | --- | --- | --- | --- | --- | --- |
| agecat | 2 | <.0001 | 0,798 | 0,737 | 0,863 | agecat 2 |
| agecat | 3 | <.0001 | 0,766 | 0,704 | 0,833 | agecat 3 |
| agecat | 4 | <.0001 | 0,669 | 0,611 | 0,732 | agecat 4 |
| PTH | 1 | 0,077 | 1,063 | 0,993 | 1,138 | PTH 1 |
| gender | 2 | 0,0188 | 0,925 | 0,866 | 0,987 | Patient gender 2 |
| REA_USC | 1 | 0,0118 | 1,147 | 1,031 | 1,277 | REA_USC: switch to REA or USC 1 |
| year | 2016 | 0,4929 | 1,026 | 0,953 | 1,104 | Output year = base year 2016 |
| year | 2017 | 0,4182 | 1,031 | 0,958 | 1,109 | Output year = base year 2017 |
| dlacte_sup3 | 1 | 0,0798 | 1,097 | 0,989 | 1,218 | dlacte_sup3: Delay between sej entry and act realization (days) recode >=3 |
| dlacte_sup3 | 2 | 0,475 | 0,959 | 0,854 | 1,076 |  |
| dlacte_sup3 | 3 | 0,3332 | 0,947 | 0,849 | 1,057 |  |
| categ_detail | Private | <.0001 | 1,258 | 1,159 | 1,366 | Detailed establishment category Private |
| typeCiment | 1 | 0,4235 | 0,971 | 0,904 | 1,043 | typeCiment 1 |
| CH_RENAL_1AN | 1 | <.0001 | 0,761 | 0,68 | 0,85 | Charlson-Renal disease 1 |
| CH_RD_1AN | 1 | 0,5155 | 1,078 | 0,859 | 1,354 | Charlson-Rheumatic disease 1 |
| CH_PVD_1AN | 1 | 0,1231 | 0,894 | 0,776 | 1,031 | Charlson-Peripheral vascular disease 1 |
| CH_PUD_1AN | 1 | 0,7661 | 0,951 | 0,685 | 1,322 | Charlson-Peptic ulcer disease 1 |
| CH_PLEGIA_1AN | 1 | 0,2204 | 1,1 | 0,944 | 1,281 | Charlson-Hemiplegia or paraplegia 1 |
| CH_MSL_1AN | 1 | 0,0071 | 1,505 | 1,117 | 2,028 | Charlson-Moderate or severe liver disease 1 |
| CH_MILD_LIVER_D_1AN | 1 | <.0001 | 1,474 | 1,215 | 1,789 | Charlson-Mild liver disease 1 |
| CH_METS_1AN | 1 | 0,0017 | 0,684 | 0,54 | 0,867 | Charlosn- Metastases 1 |
| CH_MALIGNANCY_1AN | 1 | <.0001 | 0,754 | 0,661 | 0,859 | Charlson- Any malignancy, including lymphoma and leukaemia, except malignant neoplasm of skin 1 |
| CH_HIV_1AN | 1 | 0,2643 | 1,453 | 0,754 | 2,8 | Charlson-HIV 1 |
| CH_DM_COMP_1AN | 1 | 0,126 | 0,87 | 0,728 | 1,04 | Charlson-Diabetes with chronic complication 1 |
| CH_DM_1AN | 1 | 0,1793 | 0,941 | 0,861 | 1,028 | Charlson-Diabetes without chronic complication 1 |
| CH_DEMENTIA_1AN | 1 | <.0001 | 1,236 | 1,15 | 1,329 | Charlson_dementia 1 |
| CH_CVD_1AN | 1 | 0,0014 | 0,821 | 0,728 | 0,927 | Charlson -Cerebrovascular disease 1 |
| CH_COPD_1AN | 1 | 0,6402 | 0,974 | 0,87 | 1,089 | Charlson- COPD 1 |
| CH_CHF_1AN | 1 | 0,0003 | 0,841 | 0,766 | 0,924 | Charlson- Congestive heart failure 1 |
| CH_MI_1AN | 1 | 0,0004 | 0,779 | 0,679 | 0,895 | Charlson-Myocardial infarction 1 |
| chf | 1 | 0,0003 | 0,841 | 0,766 | 0,924 | Congestive heart failure 1 |
| carit | 1 | 0,0021 | 0,896 | 0,836 | 0,961 | Cardiac arrhythmias 1 |
| valv | 1 | 0,2755 | 0,929 | 0,814 | 1,06 | Valvular disease 1 |
| pcd | 1 | 0,4281 | 1,076 | 0,897 | 1,291 | Pulmonary circulation disorders 1 |
| pvd | 1 | 0,1231 | 0,894 | 0,776 | 1,031 | Peripheral vascular disorders 1 |
| hypc | 1 | 0,1945 | 0,882 | 0,729 | 1,066 | Hypertension, complicated 1 |
| hypunc | 1 | 0,0069 | 0,92 | 0,866 | 0,977 | Hypertension, uncomplicated 1 |
| para | 1 | 0,2204 | 1,1 | 0,944 | 1,281 | Paralysis 1 |
| ond | 1 | <.0001 | 1,237 | 1,133 | 1,351 | Other neurological disorders 1 |
| cpd | 1 | 0,6402 | 0,974 | 0,87 | 1,089 | Chronic pulmonary disease 1 |
| diabunc | 1 | 0,0873 | 0,924 | 0,844 | 1,012 | Diabetes, uncomplicated 1 |
| diabc | 1 | 0,1933 | 0,894 | 0,756 | 1,058 | Diabetes, complicated 1 |
| hypothy | 1 | 0,4404 | 1,043 | 0,937 | 1,161 | Hypothyroidism 1 |
| rf | 1 | <.0001 | 0,761 | 0,681 | 0,85 | Renal failure 1 |
| ld | 1 | 0,0001 | 1,434 | 1,193 | 1,723 | Liver disease 1 |
| pud | 1 | 0,8715 | 1,044 | 0,617 | 1,767 | Peptic ulcer disease, excluding bleeding 1 |
| aids | 1 | 0,7648 | 0,861 | 0,322 | 2,299 | AIDS/HIV 1 |
| lymph | 1 | 0,0888 | 0,724 | 0,499 | 1,05 | Lymphoma 1 |
| metacanc | 1 | 0,0017 | 0,684 | 0,54 | 0,867 | Metastatic cancer 1 |
| solidtum | 1 | 0,2309 | 0,816 | 0,585 | 1,138 | Solid tumour without metastasis 1 |
| rheumd | 1 | 0,838 | 1,023 | 0,821 | 1,276 | Rheumatoid arthritis/collagen vascular diseases 1 |
| coag | 1 | 0,6626 | 0,959 | 0,796 | 1,156 | Coagulopathy 1 |
| obes | 1 | 0,0864 | 1,134 | 0,982 | 1,309 | Obesity 1 |
| wloss | 1 | 0,0891 | 0,938 | 0,871 | 1,01 | Weight loss 1 |
| fed | 1 | 0,1742 | 0,947 | 0,875 | 1,025 | Fluid and electrolyte disorders 1 |
| blane | 1 | 0,173 | 1,115 | 0,953 | 1,304 | Blood loss anaemia 1 |
| dane | 1 | 0,3136 | 0,945 | 0,846 | 1,055 | Deficiency anaemia 1 |
| alcohol | 1 | <.0001 | 1,799 | 1,605 | 2,015 | Acohol abuse 1 |
| drug | 1 | 0,2585 | 1,383 | 0,788 | 2,429 | Drug abuse 1 |
| psycho | 1 | <.0001 | 1,712 | 1,396 | 2,1 | Psychoses 1 |
| depre | 1 | 0,0008 | 1,173 | 1,068 | 1,287 | Depression 1 |
| age |  | <.0001 | 0,984 | 0,981 | 0,987 | Age in years |
| Initial_Weight_1AN | | <.0001 | 0,968 | 0,953 | 0,983 | Charlson Score |
| SC_elihauw_Van | | 0,0007 | 0,993 | 0,989 | 0,997 |  |
| Fdep09 |  | 0,1926 | 0,982 | 0,955 | 1,009 | Fdep09 - social defav index |
| duration |  | <.0001 | 1,015 | 1,012 | 1,017 | Total length of stay (in nights) |
| dlacte |  | 0,0842 | 0,99 | 0,979 | 1,001 | dlacte: Delay between sej entry and act realization (days) |
| exit_deposit_time | | <.0001 | 1,017 | 1,015 | 1,02 |  |
| IGS |  | 0,0083 | 0,99 | 0,982 | 0,997 | IGS2 |

### Multivariate model :

We present the results of two survival models with competitive risks

- CSH and SDH

Adjustment variables :

| **PTH** |
| --- |
| **agecat** |
| **gender** |
| **SC_elihauw_Van: comorb score** |
| **categ_detail** |
| **obes** |
| **Fdep09: defav social** |
| **typeCiment** |
| **year** |
| **dlacte_sup3 : dela de real of the act** |

| Dislocations | **CSH** | | | | | | **SDH fine And Gray model** | | | | |
| --- | --- | --- | --- | --- | --- | --- | --- | --- | --- | --- | --- |
| **Wording** | **Parameter** |  | **Pr > khi-2** | **Report** | **Confidence interval of the 95% hazard ratio** | | **Pr > khi-2** | **Report** | **Confidence interval of the 95% hazard ratio** | |  |
|  |  |  |  | **risk** |  |  |  | **risk** |  |  |  |
| PTH 1 | **PTH** | **1** | 0,0266 | 0,926 | 0,866 | 0,991 | 0,4173 | 0,969 | 0,898 | 1,046 |  |
| **agecat 2** | **agecat** | **2** | <.0001 | 0,831 | 0,77 | 0,897 | <.0001 | 0,826 | 0,76 | 0,897 |  |
|  |  | **3** | <.0001 | 0,791 | 0,729 | 0,858 | <.0001 | 0,774 | 0,709 | 0,846 |  |
|  |  | **4** | 0,0009 | 0,869 | 0,8 | 0,944 | <.0001 | 0,829 | 0,761 | 0,903 |  |
| **Patient gender 1** | **gender** | **1** | 0,7529 | 1,01 | 0,95 | 1,073 | 0,4576 | 0,976 | 0,914 | 1,041 |  |
| **SC_elihauw_Van** |  |  | 0,3685 | 1,002 | 0,998 | 1,005 | 0,2314 | 0,998 | 0,994 | 1,001 |  |
| **Detailed establishment category Private** | **categ_detail** | **Private** | <.0001 | 1,307 | 1,199 | 1,425 | <.0001 | 1,328 | 1,22 | 1,445 |  |
| **Obesity 1** | **obes** | **1** | 0,5226 | 0,956 | 0,832 | 1,098 | 0,7837 | 0,981 | 0,857 | 1,124 |  |
| **Fdep09 - social defav index** | **Fdep09** |  | 0,3716 | 1,012 | 0,986 | 1,038 | 0,0571 | 1,027 | 0,999 | 1,055 |  |
| **typeCiment 1** | **typeCiment** | **1** | 0,0646 | 1,064 | 0,996 | 1,135 | 0,6084 | 1,022 | 0,941 | 1,109 |  |
| **Output year = base year** | **year** | **2016** | 0,9602 | 1,002 | 0,938 | 1,07 | 0,9336 | 0,997 | 0,931 | 1,068 |  |
|  | **year** | **2017** | 0,1789 | 1,046 | 0,98 | 1,116 | 0,1973 | 1,045 | 0,977 | 1,117 |  |
| **dlacte_sup3: Delay between sej entry and act real (jrs) recode >=3 1** | **dlacte_sup3** | **1** | 0,2138 | 1,061 | 0,966 | 1,165 | 0,119 | 1,074 | 0,982 | 1,174 |  |
|  | **dlacte_sup3** | **2** | 0,9535 | 0,997 | 0,9 | 1,105 | 0,9243 | 1,005 | 0,908 | 1,113 |  |
|  | **dlacte_sup3** | **3** | 0,1339 | 0,927 | 0,84 | 1,024 | 0,1479 | 0,927 | 0,836 | 1,027 |  |

Results for PTH differ according to which method is used:

PIH is associated with a higher instantaneous risk of dislocation (for patients who have not yet died) than PTH, but this does not increase the incidence of dislocation in the 2 years following placement (because patients actually die more).

For the other factors, the results converge between the two methods ....

### Test of some interactions (on multiple model, CSH )

#### PTH*cement type: **p=0 .0178**

| **PTH*cement type** |  |  |  |  |  |
| --- | --- | --- | --- | --- | --- |
| **Effect** | **Khi-2** | **DDL** | **Pr > khi-2** | **DDL** | **Pr > khi-2** |
|  | **de Wald** |  |  | **adjusted** | **adjusted** |
| **PTH** | 9,6651 | 1 | 0,0019 | 0,9128 | 0,0016 |
| **agecat** | 36,6693 | 3 | <.0001 | 2,9799 | <.0001 |
| **gender** | 0,1053 | 1 | 0,7456 | 0,9973 | 0,7446 |
| **SC_elihauw_Van** | 0,7348 | 1 | 0,3913 | 0,9878 | 0,3868 |
| **categ_detail** | 36,641 | 1 | <.0001 | 0,5222 | <.0001 |
| **obes** | 0,4427 | 1 | 0,5058 | 0,9934 | 0,5031 |
| **Fdep09** | 0,778 | 1 | 0,3778 | 0,843 | 0,3204 |
| **typeCiment** | 0,0424 | 1 | 0,8369 | 0,8997 | 0,8019 |
| **year** | 2,385 | 2 | 0,3035 | 1,9887 | 0,3013 |
| **dlacte_sup3** | 15,5643 | 3 | 0,0014 | 2,9523 | 0,0013 |
| **PTH*cement type** | 5,5057 | 1 | 0,019 | 0,9614 | 0,0178 |
| **finess** | 654,5955 | . | . | 281,33 | <.0001 |
|  |  |  |  |  |  |
| **Pairwise: Chance reports for PTH** | | | |  |  |
| **Description** | **Point estimate** | **95% confidence interval** | |  |  |
| **PTH 0 vs 1 At typeCement=0** | 1,135 | 1,048 | 1,23 |  |  |
| **PTH 1 vs 0 At typeCement=0** | 0,881 | 0,813 | 0,954 | **Protective effect of PTH only in uncemented patients (same for revision)** | |
| **PTH 0 vs 1 At typeCement=1** | 0,977 | 0,878 | 1,087 |  |  |
| **PTH 1 vs 0 At typeCement=1** | 1,024 | 0,92 | 1,14 | NS |  |
|  |  |  |  |  |  |
| **Pairwise: Chance reports for typeCement** | | | |  |  |
| **Description** | **Point estimate** | **95% confidence interval** | |  |  |
| **typeCement 0 vs 1 At PTH=0** | 0,992 | 0,916 | 1,074 |  |  |
| **typeCement 1 vs 0 At PTH=0** | 1,008 | 0,931 | 1,092 | NS |  |
| **typeCiment 0 vs 1 At PTH=1** | 0,853 | 0,769 | 0,946 |  |  |
| **typeCement 1 vs 0 At PTH=1** | 1,172 | 1,057 | 1,3 | **Cement's deleterious effect only on PTH** | |

#### Other interactions (NS)

|  | Pval |
| --- | --- |
| **categ_detail*PTH** | 0,4183 |
| **categ_det*typeCement** | 0,1827 |
| **PTH*agecat** | 0,0807 |
| PTH*dlacte_sup3 | 0,8349 |
| **PTH*sex** | 0,4989 |

## Study of transfusion occurrence within 2 years

Crosstabulation, event type * prosthesis type

| **PTH eventTransf table** | | | |
| --- | --- | --- | --- |
| **eventTransf** | **PTH** | | |
|  | **0** | **1** | **Total** |
| **0** | 48520 | 26681 | 75201 |
|  | 50,44 | 27,74 | 78,18 |
|  | 64,52 | 35,48 |  |
|  | 75,69 | 83,18 |  |
| **1 Transfusions** | 4504 | 1792 | 6296 |
|  | 4,68 | 1,86 | 6,55 |
|  | 71,54 | 28,46 |  |
|  | 7,03 | 5,59 |  |
| **2 deaths without transfusion** | 11082 | 3605 | 14687 |
|  | 11,52 | 3,75 | 15,27 |
|  | 75,45 | 24,55 |  |
|  | 17,29 | 11,24 |  |
| **Total** | 64106 | 32078 | 96184 |
|  | 66,65 | 33,35 | 100 |

**>>** slightly **fewer transfusions for PTH** (and also fewer deaths).

### Cross-tabulated TRANSFUSION VS NON TRANSFUSION (within 2 years)

=> see **TC _ TRAFS OUI NON** tab in the Transfusion Excel document.

### Univariate models

Survival regression with competitive risks (death as a competitive event) => same method and remarks as for the analysis of recoveries.

There are two possible models which are complementary and do not measure exactly the same thing:

- Cox model, Cause specific (with Frailty model to take into account establishment as a random effect and therefore intra-establishment correlation).
- More commonly used for etiological purposes

Measures the instantaneous risk of recovery associated with covariates for patients who have not yet died => provides CSHR: Cause-specific Hazard Ratio

- Fine and Gray, (with Covs sandwich estimator to take intra-establishment correlation into account). Based on cumulative incidences, measures the association between each covariate and the cumulative incidence (note that only the direction is interpreted, not the magnitude of the effect) = > **more commonly used for prediction.**

#### Summary of univariate hazard ratios (**CSH** model)

| **Parameter** | **ClassVal0** | **ProbChiSq** | **HazardRatio** | **HRLowerCL** | **HRUpperCL** | **Label** |
| --- | --- | --- | --- | --- | --- | --- |
| agecat | 2 | 0,0000 | 1,330 | 1,232 | 1,436 | agecat 2 |
| agecat | 3 | 0,0000 | 1,518 | 1,404 | 1,640 | agecat 3 |
| agecat | 4 | 0,0000 | 1,693 | 1,567 | 1,830 | agecat 4 |
| PTH | 1 | 0,0000 | 0,839 | 0,787 | 0,894 | PTH 1 |
| gender | 2 | 0,0025 | 0,919 | 0,870 | 0,971 | Patient gender 2 |
| REA_USC | 1 | 0,0000 | 1,939 | 1,778 | 2,114 | REA_USC: switch to REA or USC 1 |
| year | 2016 | 0,0209 | 0,932 | 0,877 | 0,989 | Output year = base year 2016 |
| year | 2017 | 0,0000 | 0,867 | 0,815 | 0,923 | Output year = base year 2017 |
| dlacte_sup3 | 1 | 0,7660 | 0,986 | 0,902 | 1,079 | dlacte_sup3: Delay between sej entry and act realization (days) recode >=3 |
| dlacte_sup3 | 2 | 0,0574 | 1,099 | 0,997 | 1,212 |  |
| dlacte_sup3 | 3 | 0,0000 | 1,241 | 1,131 | 1,362 |  |
| categ_detail | Private | 0,0174 | 1,274 | 1,043 | 1,555 | Detailed establishment category Private |
| typeCiment | 1 | 0,0000 | 1,342 | 1,256 | 1,433 | typeCiment 1 |
| CH_RENAL_1AN | 1 | 0,0000 | 1,823 | 1,698 | 1,957 | Charlson-Renal disease 1 |
| CH_RD_1AN | 1 | 0,0366 | 1,216 | 1,012 | 1,460 | Charlson-Rheumatic disease 1 |
| CH_PVD_1AN | 1 | 0,0000 | 1,453 | 1,314 | 1,606 | Charlson-Peripheral vascular disease 1 |
| CH_PUD_1AN | 1 | 0,0000 | 1,935 | 1,572 | 2,381 | Charlson-Peptic ulcer disease 1 |
| CH_PLEGIA_1AN | 1 | 0,3686 | 1,063 | 0,931 | 1,214 | Charlson-Hemiplegia or paraplegia 1 |
| CH_MSL_1AN | 1 | 0,0000 | 2,693 | 2,177 | 3,331 | Charlson-Moderate or severe liver disease 1 |
| CH_MILD_LIVER_D_1AN | 1 | 0,0000 | 1,833 | 1,563 | 2,148 | Charlson-Mild liver disease 1 |
| CH_METS_1AN | 1 | 0,0000 | 1,698 | 1,481 | 1,948 | Charlosn- Metastases 1 |
| CH_MALIGNANCY_1AN | 1 | 0,0000 | 1,755 | 1,619 | 1,903 | Charlson- Any malignancy, including lymphoma and leukaemia, except malignant neoplasm of skin 1 |
| CH_HIV_1AN | 1 | 0,6162 | 1,183 | 0,613 | 2,281 | Charlson-HIV 1 |
| CH_DM_COMP_1AN | 1 | 0,0000 | 1,401 | 1,235 | 1,589 | Charlson-Diabetes with chronic complication 1 |
| CH_DM_1AN | 1 | 0,0001 | 1,150 | 1,071 | 1,234 | Charlson-Diabetes without chronic complication 1 |
| CH_DEMENTIA_1AN | 1 | 0,0481 | 1,064 | 1,001 | 1,132 | Charlson_dementia 1 |
| CH_CVD_1AN | 1 | 0,0000 | 1,216 | 1,112 | 1,328 | Charlson -Cerebrovascular disease 1 |
| CH_COPD_1AN | 1 | 0,0001 | 1,192 | 1,089 | 1,304 | Charlson- COPD 1 |
| CH_CHF_1AN | 1 | 0,0000 | 1,642 | 1,540 | 1,752 | Charlson- Congestive heart failure 1 |
| CH_MI_1AN | 1 | 0,0000 | 1,481 | 1,352 | 1,622 | Charlson-Myocardial infarction 1 |
| chf | 1 | 0,0000 | 1,642 | 1,540 | 1,752 | Congestive heart failure 1 |
| carit | 1 | 0,0000 | 1,428 | 1,353 | 1,507 | Cardiac arrhythmias 1 |
| valv | 1 | 0,0000 | 1,765 | 1,616 | 1,927 | Valvular disease 1 |
| pcd | 1 | 0,0000 | 1,553 | 1,360 | 1,774 | Pulmonary circulation disorders 1 |
| pvd | 1 | 0,0000 | 1,453 | 1,314 | 1,606 | Peripheral vascular disorders 1 |
| hypc | 1 | 0,0000 | 1,522 | 1,332 | 1,739 | Hypertension, complicated 1 |
| hypunc | 1 | 0,0000 | 1,305 | 1,240 | 1,374 | Hypertension, uncomplicated 1 |
| para | 1 | 0,3686 | 1,063 | 0,931 | 1,214 | Paralysis 1 |
| ond | 1 | 0,0361 | 1,088 | 1,005 | 1,177 | Other neurological disorders 1 |
| cpd | 1 | 0,0001 | 1,192 | 1,089 | 1,304 | Chronic pulmonary disease 1 |
| diabunc | 1 | 0,0003 | 1,144 | 1,065 | 1,230 | Diabetes, uncomplicated 1 |
| diabc | 1 | 0,0000 | 1,349 | 1,196 | 1,522 | Diabetes, complicated 1 |
| hypothy | 1 | 0,0566 | 1,090 | 0,998 | 1,191 | Hypothyroidism 1 |
| rf | 1 | 0,0000 | 1,824 | 1,699 | 1,958 | Renal failure 1 |
| ld | 1 | 0,0000 | 1,906 | 1,647 | 2,207 | Liver disease 1 |
| pud | 1 | 0,0757 | 1,421 | 0,964 | 2,094 | Peptic ulcer disease, excluding bleeding 1 |
| aids | 1 | 0,5648 | 1,227 | 0,612 | 2,461 | AIDS/HIV 1 |
| lymph | 1 | 0,0000 | 2,266 | 1,859 | 2,763 | Lymphoma 1 |
| metacanc | 1 | 0,0000 | 1,698 | 1,481 | 1,948 | Metastatic cancer 1 |
| solidtum | 1 | 0,0001 | 1,538 | 1,237 | 1,913 | Solid tumour without metastasis 1 |
| rheumd | 1 | 0,0696 | 1,179 | 0,987 | 1,407 | Rheumatoid arthritis/collagen vascular diseases 1 |
| coag | 1 | 0,0000 | 1,952 | 1,731 | 2,201 | Coagulopathy 1 |
| obes | 1 | 0,5252 | 1,041 | 0,919 | 1,180 | Obesity 1 |
| wloss | 1 | 0,0000 | 1,487 | 1,401 | 1,577 | Weight loss 1 |
| fed | 1 | 0,0000 | 1,544 | 1,453 | 1,641 | Fluid and electrolyte disorders 1 |
| blane | 1 | 0,0000 | 3,308 | 3,010 | 3,635 | Blood loss anaemia 1 |
| dane | 1 | 0,0000 | 2,247 | 2,093 | 2,413 | Deficiency anaemia 1 |
| alcohol | 1 | 0,0077 | 1,172 | 1,043 | 1,317 | Acohol abuse 1 |
| drug | 1 | 0,9492 | 1,019 | 0,577 | 1,798 | Drug abuse 1 |
| psycho | 1 | 0,8305 | 1,023 | 0,830 | 1,261 | Psychoses 1 |
| depre | 1 | 0,0008 | 1,145 | 1,058 | 1,239 | Depression 1 |
| age |  | 0,0000 | 1,020 | 1,017 | 1,022 | Age in years |
| Initial_Weight_1AN |  | 0,0000 | 1,116 | 1,105 | 1,127 | Charlson Score |
| SC_elihauw_Van |  | 0,0000 | 1,035 | 1,032 | 1,038 |  |
| Fdep09 |  | 0,1534 | 0,980 | 0,954 | 1,007 | Fdep09 - social defav index |
| duration |  | 0,0000 | 1,019 | 1,017 | 1,021 | Total length of stay (in nights) |
| dlacte |  | 0,0000 | 1,026 | 1,021 | 1,032 | dlacte: Delay between sej entry and act realization (days) |
| exit_deposit_time |  | 0,0000 | 1,018 | 1,016 | 1,021 |  |
| IGS |  | 0,7171 | 1,001 | 0,996 | 1,007 | IGS2 |

### Multivariate model :

We present the results of two survival models with competitive risks

- CSH and SDH

Adjustment variables :

| **PTH** |
| --- |
| **agecat** |
| **gender** |
| **SC_elihauw_Van: comorb score** |
| **categ_detail** |
| **obes** |
| **Fdep09: defav social** |
| **typeCiment** |
| **year** |
| **dlacte_sup3 : dela de real of the act** |

| **TRANSFUSION modeling** | **CSH Model** |  |  |  |  |  |  | **SDH** |  |  |  |
| --- | --- | --- | --- | --- | --- | --- | --- | --- | --- | --- | --- |
|  | **Parameter** |  | **Pr > khi-2** | **Report** | **Confidence interval of the 95% hazard ratio** | |  | **Pr > khi-2** | **Report** | **Confidence interval of the 95% hazard ratio** | |
|  |  |  |  | **risk** |  |  |  |  | **risk** |  |  |
| **PTH 1** | **PTH** | **1** | 0,0955 | 1,062 | 0,99 | 1,139 |  | 0,1435 | 0,895 | 0,771 | 1,038 |
| **agecat** | **agecat** | **2** | <.0001 | 1,304 | 1,203 | 1,413 |  | <.0001 | 1,213 | 1,114 | 1,322 |
|  | **agecat** | **3** | <.0001 | 1,463 | 1,345 | 1,591 |  | <.0001 | 1,302 | 1,198 | 1,414 |
|  | **agecat** | **4** | <.0001 | 1,631 | 1,498 | 1,776 |  | <.0001 | 1,399 | 1,274 | 1,535 |
| **Patient gender 1** | **gender** | **1** | 0,1172 | 1,046 | 0,989 | 1,106 |  | 0,1112 | 1,051 | 0,989 | 1,118 |
|  | **SC_elihauw_Van** |  | <.0001 | 1,033 | 1,03 | 1,036 |  | <.0001 | 1,025 | 1,02 | 1,029 |
| **Detailed establishment category Private** | **categ_detail** | **Private** | 0,0355 | 1,243 | 1,015 | 1,523 |  | 0,4959 | 1,084 | 0,859 | 1,367 |
| **Obesity 1** | **obes** | **1** | 0,0913 | 1,115 | 0,983 | 1,266 |  | 0,0207 | 1,165 | 1,024 | 1,326 |
| **Fdep09 - social defav index** | **Fdep09** |  | 0,1861 | 0,982 | 0,955 | 1,009 |  | 0,979 | 0,999 | 0,948 | 1,053 |
| **typeCiment 1** | **typeCiment** | **1** | **<.0001** | **1,272** | **1,19** | **1,36** |  | **0,0053** | **1,256** | **1,07** | **1,474** |
| **Year of release** | **year** | **2016** | 0,002 | 0,909 | 0,855 | 0,966 |  | 0,0534 | 0,914 | 0,834 | 1,001 |
|  | **year** | **2017** | <.0001 | 0,843 | 0,792 | 0,897 |  | 0,0084 | 0,846 | 0,747 | 0,958 |
| **dlacte_sup3: Delay between sej entry and act real (jrs) recode >=3 1** | **dlacte_sup3** | **1** | 0,8963 | 0,994 | 0,908 | 1,088 |  | 0,7226 | 0,974 | 0,84 | 1,128 |
|  | **dlacte_sup3** | **2** | 0,244 | 1,06 | 0,961 | 1,17 |  | 0,9492 | 1,005 | 0,858 | 1,178 |
|  | **dlacte_sup3** | **3** | 0,0427 | 1,102 | 1,003 | 1,211 |  | 0,6583 | 1,039 | 0,878 | 1,229 |

When adjusted, we observe that there are no differences for PTH but according to cement YES=> more transfusions for cemented prostheses.

The two models do not reach the same conclusions for the type of establishment variable.

Being cared for in a private hospital increases the instantaneous risk of transfusion, but not the incidence **(they die less).**

# Study of the transition to REA - USC

**Cross-tabulated with PTH / Typeciment**

|  | **REA_USC: switch to REA or USC** | | | | | |
| --- | --- | --- | --- | --- | --- | --- |
|  | **0** | | | **1** | | |
|  | **N** | **%** | **Tx** | **N** | **%** | **Tx** |
| **PTH** | 59593 | 66,9 | 92,96 | 4513 | 63,53 | 7,04 |
| **0** |  |  |  |  |  |  |
| **1** | 29487 | 33,1 | 91,92 | 2591 | 36,47 | 8,08 |
| **typeCiment** | 61283 | 68,8 | 92,97 | 4631 | 65,19 | 7,03 |
| **0** |  |  |  |  |  |  |
| **1** | 27797 | 31,2 | 91,83 | 2473 | 34,81 | 8,17 |

**>>** slightly **more PTH and typeciment switch to REA /USC.**

### Cross table REA /USC VS NON REA /USC

=> see the **TC_PASSAGE REA** tab in the REA Excel doc.

=> univariate stats => cf OR in excel doc on REA passages

Cross-tabulation=> transition to rea according to pth and type of establishment

| **Detailed plant category** | **PTH** | **REA_USC: switch to REA or USC** | |
| --- | --- | --- | --- |
|  |  | **1** | |
|  |  | **N** | **%** |
| **PUBLIC** | **0** | 2812 | 5,53 |
|  | **1** | 1107 | 5,32 |
| **Private** | **0** | 1800 | 12,4 |
|  | **1** | 1520 | 12,96 |

In private establishments, PTH patients are often hospitalized in REA, whereas the opposite is true in public establishments. Public.

### Principal univariate ORs (GLM model - with random finess)

|  |  |  | **OR** | **Lower** | **Upper** |
| --- | --- | --- | --- | --- | --- |
| agecat | 2 | 1 | 1,204 | 1,113 | 1,303 |
|  | 3 | 1 | 1,281 | 1,181 | 1,389 |
|  | 4 | 1 | 1,307 | 1,205 | 1,418 |
| PTH | *1 | 0 | 0,783 | 0,732 | 0,839 |
| gender | 2 | 1 | 0,473 | 0,447 | 0,501 |
|  |  |  |  |  |  |
| year | 2016 | 2015 | 0,967 | 0,905 | 1,033 |
|  | 2017 | 2015 | 0,923 | 0,864 | 0,987 |
| dlacte_sup3 | 1 | 0 | 0,975 | 0,878 | 1,081 |
|  | 2 | 0 | 1,296 | 1,161 | 1,448 |
|  | 3 | 0 | 2,056 | 1,853 | 2,281 |
| categ_detail | Private | PUBLIC | 2,31 | 1,802 | 2,96 |
| typeCiment | 1 | 0 | 1,311 | 1,222 | 1,407 |

*** here, the OR for REA is inverted in relation to the raw figures, because in fact, PTHs are more often performed in private establishments, which hospitalize more REA patients (whereas the opposite is true for public establishments).**

### Results muliple/adjusted model

| **Estimated odds ratio** | | | |  |
| --- | --- | --- | --- | --- |
|  | **Estimate** | **95% confidence interval** | | **Pr > F** |
| **PTH** | 0,995 | 0,921 | 1,076 | 0,9094 |
| **agecat** | 1,111 | 1,019 | 1,211 | 0,0017 |
|  | 1,162 | 1,061 | 1,272 |  |
|  | 1,19 | 1,085 | 1,306 |  |
| **Gender** | 1,673 | 1,574 | 1,778 | <.0001 |
| **SC_elihauw_Van** | 1,092 | 1,088 | 1,095 | <.0001 |
| **Fdep09** | 1,06 | 1,027 | 1,094 | <.0001 |
| Private | 2,823 | 2,157 | 3,695 | <.0001 |
| **Obesity** | 2,44 | 2,15 | 2,769 | 0,0003 |
| **typeCiment** | 1,254 | 1,164 | 1,35 | <.0001 |
| **Year** | 0,936 | 0,873 | 1,004 | 0,0011 |
|  | 0,876 | 0,816 | 0,94 |  |
| **dlacte_sup3:** | 0,963 | 0,863 | 1,074 | <.0001 |
|  | 1,146 | 1,02 | 1,286 |  |
|  | 1,448 | 1,298 | 1,616 |  |

#### Search for interactions (in the multiple model)

| PTH*cement type | P val= 0.2805 |
| --- | --- |

| categ_detail*PTH | P val = | <.0001 |
| --- | --- | --- |

#### PTH vs PIH by type of facility

|  | OR and 95% CI | | |
| --- | --- | --- | --- |
| **categ_detail Private** | 0,8 | 0,712 | 0,898 |
| **categ_detail PUBLIC** | 1,17 | 1,06 | 1,291 |

In private establishments, adjusted for other factors, there was less risk of transfer to REA for PTH vs PIH, whereas in public establishments the opposite was true, i.e. more risk of transfer to REA/ USC if PTH vs PIH.

#### Type of facility by PTH or PIH: OR private vs public

|  | **OR and 95% CI** | | |
| --- | --- | --- | --- |
| **PTH 1** | 2,273 | 1,71 | 3,021 |
| **PTH 0** | 3,324 | 2,521 | 4,383 |

Overall, greater risk of transition to REA in private vs. public sector (adjusted for other factors in the model); but greater effect for PIH

| categ_det*typeCement | P val : | 0,9027 |
| --- | --- | --- |

# Summary table of global pop analyses _ **PTH vs PIH** => main outcomes (in blue, adjusted and main results)

| **WITHOUT MATCHING** | |  |  |  |  |  |  |  |  |  |  |  |  |  |
| --- | --- | --- | --- | --- | --- | --- | --- | --- | --- | --- | --- | --- | --- | --- |
|  | **DC 2ANS** |  | **COX FM Univ** | | **COX FM Multiv** | |  |  |  |  |  | |  |  |
|  | **N** | **%** | **P val** | **HR** | **P val** | **HR 95% IC** |  |  |  |  |  | |  |  |
| **PTH** | 3991 | 12.44 | <.0001 | 0,568 [0,546-0,591] | <.0001 | 0,762 [0,731-0,795] |  |  |  |  |  | |  |  |
| **PIH** | 12247 | 19.1 |  |  | ***Interaction pth* typeciment, p=0.0003*** | |  |  |  |  |  | |  |  |
|  |  |  |  |  | *In cement patients, PTH vs PIH -Cox Model* | |  |  |  |  |  | |  |  |
|  |  |  |  |  | ***<0,05*** | *0,835 [0,782-0,892]* |  |  |  |  |  | |  |  |
|  |  |  |  |  | *In non-cemented patients, PTH vs PIH* | |  |  |  |  |  | |  |  |
|  |  |  |  |  | ***<0,05*** | *0,725 [0,689 - 0,763]* |  |  |  |  |  | |  |  |
|  |  |  |  |  | ***Interaction pth*categ , p=0.0385*** |  |  |  |  |  |  | |  |  |
|  |  |  |  |  |  |  |  |  |  |  |  | |  |  |
|  | **RESET 2 YEARS** |  | **COX FM -csh Univ** | | **COX FM CSH Multiv** | |  |  | **COX multiple - SDH Fine and Gray** | | | | |  |
| **PTH** | 1519 | 4.74 | 0.077 | 1,063 [0.993-1.138] - CSH | **0.0168** | **0.914 [0.849-0.984] - CSH** |  |  | *0.3109* | *0,959 [0,884 - 1,04]* | NS | |  |  |
| **PIH** | 2828 | 4.41 |  |  | ***PTH*typeciment interaction,p=0.028*** | |  |  |  |  |  | |  |  |
|  |  |  |  |  | In cement patients, PTH vs PIH - CSH Model | |  |  |  |  |  | |  |  |
|  |  |  |  |  | NS | 1,017 [0,901-1,148] |  |  |  |  |  | |  |  |
|  |  |  |  |  | In non-cemented patients, PTH vs PIH | |  |  |  |  |  | |  |  |
|  |  |  |  |  | ***<0,05*** | ***0,87 [0,798 - 0,948]*** |  |  |  |  |  | |  |  |
|  |  |  |  |  |  |  |  |  |  |  |  | |  |  |
|  | **DISLOCATIONS 2 YEARS** |  | **COX FM -csh Univ** | | **COX FM CSH Multiv** | |  |  | **COX multiple - SDH Fine and Gray** | | | | |  |
| **PTH** | 1904 | 5.94 | 0.6703 | 1.014 [0.953 - 1.078] -CSH | **0.0266** | **0,926 [0,866-0,991]** |  |  | *0.4173* | *0,969 [0,898 - 1,046]* | NS | |  |  |
| **PIH** | 3647 | 5.69 |  |  | ***PTH*typeciment interaction,p=0.0178*** |  |  |  |  |  |  | |  |  |
|  |  |  |  |  | *In cement patients, PTH vs PIH - CSH Model* | |  |  |  |  |  | |  |  |
|  |  |  |  |  | *NS* | *1,024 [0,92- 1,14]* |  |  |  |  |  | |  |  |
|  |  |  |  |  | *In non-cemented patients, PTH vs PIH* | |  |  |  |  |  | |  |  |
|  |  |  |  |  | *<0,05* | *0,881 [0,813 - 0,954]* |  |  |  |  |  | |  |  |
|  |  |  |  |  |  |  |  |  |  |  |  | |  |  |
|  | **TRANSFUSIONS 2 YEARS** |  | **COX FM -csh Univ** | | **COX FM CSH Multiv** | |  |  | **COX multiple - SDH Fine and Gray** | | | |  |  |
| **PTH** | 1792 | 5.59 | <.0001 | 0,839 [0,787 - 0,894] | 0.0955 | 1,062 [0,99 - 1,139] |  |  | *0.1435* | *0,895 [0,771 -1,038]* |  | |  |  |
| **PIH** | 4504 | 7.03 |  |  | ***PTH*typeciment interaction,p=0.016*** |  |  |  |  |  |  | |  |  |
|  |  |  |  |  | *In cement patients, PTH vs PIH - CSH Model* | |  |  |  |  |  | |  |  |
|  |  |  |  |  |  | ***1,165 [1,051- 1,291]*** |  |  |  |  |  | |  |  |
|  |  |  |  |  | *In non-cemented patients, PTH vs PIH* | |  |  |  |  |  | |  |  |
|  |  |  |  |  |  | 1,002 [0,92 - 1,091] |  |  |  |  |  | |  |  |
|  |  |  |  |  | ***PTH*prive interaction ,p=0.0472*** |  |  |  |  |  |  | |  |  |
|  |  |  |  |  | ** inter with PTH signif / + risk in private if PIH* | |  |  |  |  |  | |  |  |
|  | **CHANGEOVER TO REA/USC** |  | **GLM logit Model (random finess) - univ** | | **GLM logit Model (random finess) - Multiv** | | |  |  |  |  | |  |  |
| **PTH** | 2591 | 8.08 | <.0001 | 0,783 [0,732 - 0,859] | 0.9094 | 0,995 [0,921 - 1,076] |  |  |  |  |  | |  |  |
| **PIH** | 4513 | 7.04 |  |  |  |  |  |  |  |  |  | |  |  |
|  |  |  |  |  | Weird interaction with categ_detail | |  |  |  |  |  | |  |  |

# Summary table of global pop analyses _ **Cement vs non-cement** => main outcomes (in blue, adjusted and main results)

| **WITHOUT MATCHING** | | |  |  |  |  |  |  |  |  |  |  |
| --- | --- | --- | --- | --- | --- | --- | --- | --- | --- | --- | --- | --- |
|  | **DC 2ANS** |  | **COX FM Univ** | | **COX FM Multiv** | |  |  |  |  |  |  |
| **typeCiment** | **n** | **%** | **p val** | **HR** | **p val** | **HR** |  |  |  |  |  |  |
| **0** | 10651 | 16.16 |  |  |  |  |  |  |  |  |  |  |
| **1** | 5587 | 18.46 | <.0001 | 1.108 [1.073 -1.145] | **<.0001** | **1.107[1.067 - 1.149]** |  |  |  |  |  |  |
|  |  |  |  |  |  |  |  |  |  |  |  |  |
|  | **RESET 2 YEARS** |  | **COX FM -csh Univ** | | **COX FM CSH Multiv** | |  |  | **COX multiple - SDH Fine and Gray** | |  |  |
| **typeCiment** | **n** | **%** | **p val** | **HR** | **P val** | **HR** |  |  | **P val** | **HR** |  |  |
| **0** | 3004 | 4.56 |  |  |  |  |  |  |  |  |  |  |
| **1** | 1343 | 4.44 | 0.4235 | 0.971 [0.904-1.043] | 0.5256 | 0.977 [0.909 -1.05] |  |  | 0.2432 | 0.947 [0.865 - 1.038] |  |  |
|  |  |  |  |  |  |  |  |  |  |  |  |  |
|  | **DISLOCATIONS 2 YEARS** |  | **COX FM -csh Univ** | | **COX FM CSH Multiv** | |  |  | **COX multiple - SDH Fine and Gray** | |  |  |
| **typeCiment** | **n** | **%** | **p val** | **HR** | **p val** | **HR** |  |  | **P val** | **HR** |  |  |
| **0** | 3762 | 5.71 |  |  |  |  |  |  |  |  |  |  |
| **1** | 1789 | 5.91 | 0.0695 | 1.062 [0.995 - 1.134] | 0.0646 | 1.064 [0.996 -1.135] |  |  | 0.6084 | 1.022 [0.941 -1.109] |  |  |
|  |  |  |  |  | ***PTH*typeciment interaction,p=0.016*** | |  |  |  |  |  |  |
|  |  |  |  |  | ***In HSP, cement vs. non-cement*** | |  |  |  |  |  |  |
|  |  |  |  |  |  | 1.008 [0.931 - 1.092] NS |  |  |  |  |  |  |
|  |  |  |  |  | ***Cement vs. non-cement PTH*** | |  |  |  |  |  |  |
|  |  |  |  |  |  | 1.172 [1.057 - 1.3] |  |  |  |  |  |  |
|  |  |  |  |  | **Cement's deleterious effect only on PTH** | | |  |  |  |  |  |
|  |  |  |  |  | * NS in an. Sensitivity pop matched | |  |  |  |  |  |  |
|  | **TRANSFUSIONS 2 YEARS** |  | **COX FM -csh Univ** | | **COX FM CSH Multiv** | |  |  | **COX multiple - SDH Fine and Gray** | |  |  |
| **typeCiment** | **n** | **%** | **p val** | **HR** | **p val** | **HR** |  |  | **P val** | **HR** |  |  |
| **0** | 3928 | **5.96** |  |  |  |  |  |  |  |  |  |  |
| **1** | 2368 | **7.82** | <.0001 | 0.839 [0.787 - 0.894] | <.0001 | 1.272 [1.19 - 1.36] |  |  | 0.0053 | 1.256 [1.047 -1.474 ] |  |  |
|  |  |  |  |  |  |  |  |  |  |  |  |  |
|  | **CHANGEOVER TO REA/USC** |  | **GLM logit Model (random finess) - univ** | | **GLM logit Model (random finess) - Multiv** | |  |  |  |  |  |  |
| **typeCiment** | **n** | **%** | **p val** | **HR** | **p val** | **HR** |  |  |  |  |  |  |
| **0** | 4631 | 7.03 |  |  |  |  |  |  |  |  |  |  |
| **1** | 2473 | 8.17 | <.0001 | 1.311 [1.222- 1.407] | <.0001 | 1.254 [1.164 -1.35] |  |  |  |  |  |  |

**In a nutshell:**

If we consider the CSH method for taking CRisk into account, we conclude:

- more deaths, dislocations for PIH vs PTH

- no diff on transfusion and no REA passages

=> I won't talk about interactions specifically (because an. Sensitivity (Matching) don't find them

=> same results if sensitivity year on pop. Matched

- more deaths, transfusions and REA visits for cemented vs. non-cemented = more deaths, transfusions and REA visits for cemented vs. non-cemented = more deaths, transfusions and REA visits for cemented vs. non-cemented

- no diff on recoveries and dislocations

=> I won't talk about interactions specifically (because an. Sensitivity (Matching) don't find them

=> same results if an. Sensitivity on matched population except for death where it is NS.

**To be done: See for dual mobility....**

***Test whether results are robust with continuous ageing during adjustment => ok overall yes***

**Following the telephone meeting of Nov 13 to do :**

- For mortality: redo with categorized fdep
  - OK DON'T CHANGE ANYTHING
- See if we can add diabetes to the list of comorbidities.
  - OK DOES NOT CHANGE ANYTHING (for death)
- Describe revision according to etiology => sepsis / mechanical complications / fractures

OK

- Review the need to stratify the analysis according to the different types of recovery ....
  - OK different models made according to the type of complic and it changes ...
- The result on transfusion and cement is strange => more transfusions for cemented ????
- Repeat PS score for cement => if different results => compare the two populations before/after matching

For dc the results are maintained... more deaths if cemented

- Effect size to be released ??? I don't know ....
- Passage to rea/usc= OR univ bizarre for PTH as not going in the right direction
